# Inference of Infectious Disease Transmission through a Relaxed Bottleneck Using Multiple Genomes Per Host

**DOI:** 10.1093/molbev/msad288

**Published:** 2024-01-03

**Authors:** Jake Carson, Matt Keeling, David Wyllie, Paolo Ribeca, Xavier Didelot

**Affiliations:** Mathematics Institute, University of Warwick, Coventry CV4 7AL, UK; School of Life Sciences, University of Warwick, Coventry CV4 7AL, UK; Zeeman Institute for Systems Biology and Infectious Disease Epidemiology Research (SBIDER), University of Warwick, Coventry CV4 7AL, UK; Mathematics Institute, University of Warwick, Coventry CV4 7AL, UK; School of Life Sciences, University of Warwick, Coventry CV4 7AL, UK; Zeeman Institute for Systems Biology and Infectious Disease Epidemiology Research (SBIDER), University of Warwick, Coventry CV4 7AL, UK; UK Health Security Agency, London NW9 5EQ, UK; UK Health Security Agency, London NW9 5EQ, UK; School of Life Sciences, University of Warwick, Coventry CV4 7AL, UK; Zeeman Institute for Systems Biology and Infectious Disease Epidemiology Research (SBIDER), University of Warwick, Coventry CV4 7AL, UK; Department of Statistics, University of Warwick, Coventry CV4 7AL, UK

**Keywords:** genomic epidemiology, transmission analysis, infectious disease outbreak, within-host diversity and evolution

## Abstract

In recent times, pathogen genome sequencing has become increasingly used to investigate infectious disease outbreaks. When genomic data is sampled densely enough amongst infected individuals, it can help resolve who infected whom. However, transmission analysis cannot rely solely on a phylogeny of the genomes but must account for the within-host evolution of the pathogen, which blurs the relationship between phylogenetic and transmission trees. When only a single genome is sampled for each host, the uncertainty about who infected whom can be quite high. Consequently, transmission analysis based on multiple genomes of the same pathogen per host has a clear potential for delivering more precise results, even though it is more laborious to achieve. Here, we present a new methodology that can use any number of genomes sampled from a set of individuals to reconstruct their transmission network. Furthermore, we remove the need for the assumption of a complete transmission bottleneck. We use simulated data to show that our method becomes more accurate as more genomes per host are provided, and that it can infer key infectious disease parameters such as the size of the transmission bottleneck, within-host growth rate, basic reproduction number, and sampling fraction. We demonstrate the usefulness of our method in applications to real datasets from an outbreak of *Pseudomonas aeruginosa* amongst cystic fibrosis patients and a nosocomial outbreak of *Klebsiella pneumoniae*.

## Introduction

Pathogen genomic data has transformed our understanding of the epidemiology of infectious diseases, whether they are caused by viruses (Grenfell et al. 2004; Pybus and Rambaut 2009) or bacteria (Didelot et al. 2012; Gardy and Loman 2018). Most applications concern large-scale pathogen populations, for example to estimate their demographic history (Pybus et al. 2001; Ho and Shapiro 2011) or the way that their ancestry relates to features of geography (Lemey et al. 2009; De Maio et al. 2015), epidemiology (Volz et al. 2013; Rasmussen et al. 2014), or host population (Mather et al. 2013; Dearlove et al. 2016). Genomic data can however also be useful to perform much finer inference, down to the level of transmission analysis which attempts to reconstruct who infected whom within an outbreak (Cottam et al. 2008; Jombart et al. 2011). Phylogenetic methods have a long successful history and can reconstruct the genealogy of a set of genomes given their sequences (Yang and Rannala 2012; Kapli et al. 2020). However, a phylogenetic tree is not identical to a transmission tree (Pybus and Rambaut 2009; Jombart et al. 2011; Romero-Severson et al. 2014). In particular, the nodes in a phylogenetic tree do not correspond to transmission events, but rather to lineages diverging during the evolutionary process that takes places within a host (Didelot et al. 2016). Several methods have therefore been developed over the past few years specifically aimed at the reconstruction of a transmission tree (Duault et al. 2022). Examples include SeqTrack (Jombart et al. 2011), outbreaker (Jombart et al. 2014), beastlier (Hall et al. 2015), bitrugs (Worby et al. 2016), SCOTTI (De Maio et al. 2016), phybreak (Klinkenberg et al. 2017), outbreaker2 (Campbell et al. 2018), and TiTUS (Sashittal and El-Kebir 2020).

Here, we focus on one such method for transmission analysis called TransPhylo, which is based on coloring the branches of a dated phylogeny to reveal the transmission tree (Didelot et al. 2014). There are many software tools that can be used to construct such a dated phylogeny, for example BEAST (Suchard et al. 2018), BEAST2 (Bouckaert et al. 2019), BactDating (Didelot et al. 2018), treedater (Volz and Frost 2017), and TreeTime (Sagulenko et al. 2018). An advantage of the TransPhylo coloring approach is that it separates the initial phylogenetic reconstruction from its epidemiological interpretation, which improves computational efficiency and therefore scalability (Didelot and Parkhill 2022). Furthermore, the original TransPhylo model (Didelot et al. 2014) has been extended to deal with both partially sampled and ongoing outbreaks (Didelot et al. 2017). Consequently, TransPhylo is a flexible and versatile software to perform transmission analysis using pathogen genomic data (Didelot et al. 2021).

Following infection, many pathogens evolve within hosts on a time scale that is relevant to transmission analysis (Lieberman et al. 2011; Bryant et al. 2013; Biek et al. 2015; Grote and Earl 2022). Consequently, when information is available about the within-host pathogen diversity, this can help clarify who infected whom (Didelot et al. 2016; Leitner 2019). This information can come in 2 forms: either heterogeneities in the genomic sequencing of a single clinical sample, or genomic sequencing of multiple separate clinical samples. Genetic heterogeneities within a sample are relatively easy to survey, and a few methods have been developed recently with the specific aim of exploiting this type of data to help infer transmission (De Maio et al. 2018; Wymant et al. 2018; Torres Ortiz et al. 2023). However, this approach is based on the analysis of short sequencing reads individually which can be difficult and error-prone; additionally the clinical sample may not represent the full within-host diversity of the pathogen when it was collected, and it does not contain any information about evolution or changes of diversity over time in the within-host pathogen population. The alternative approach of sequencing several clinical samples can provide a more thorough and reliable overview of the within-host diversity and evolution, especially if the samples are taken from multiple body sites and/or at different points in time. Examples of such studies have been carried on infection with *Staphylococcus aureus* (Young et al. 2012), *Helicobacter pylori* (Didelot et al. 2013), or *Streptococcus pneumoniae* (Tonkin-Hill et al. 2022). Existing methods that can incorporate such data include beastlier (Hall et al. 2015), bitrugs (Worby et al. 2016), SCOTTI (De Maio et al. 2016), phyloscanner (Hall et al. 2019), and TiTUS (Sashittal and El-Kebir 2020).

In principle, integrating multiple genomes into a joint model of phylogenetic and transmission trees, such as TransPhylo, is possible by having as many leaves in the phylogenetic tree as there are samples (Didelot et al. 2016; Leitner 2019). However, this poses a significant number of theoretical challenges to overcome, which is why TransPhylo was not previously able to use more than 1 genome per host (Didelot et al. 2017; Xu et al. 2020). Furthermore, TransPhylo previously assumed a complete transmission bottleneck to simplify the relationship between transmission and phylogenetic trees (Didelot et al. 2014), but this assumption has been disproved in some pathogens. Here, we present a solution to these issues, which leads us to formulate an extended version of the TransPhylo model, inference methodology, and software, so that any number of genomes per host can be used as input of a transmission analysis that does not assume a complete transmission bottleneck.

## New Approaches

We extend the latest TransPhylo framework (Didelot et al. 2017) to perform inference of infectious disease transmission through a relaxed bottleneck using multiple genomes per host, which may be sampled contemporaneously or longitudinally, or in any combination of both. The model in TransPhylo has 3 basic ingredients which we detail below, before explaining the changes needed to deal with multiple samples per host. Firstly, a coalescent model with constant population size and temporally offset leaves (Drummond et al. 2002) to represent the within-host evolution. Secondly, a branching process transmission model in which individuals are sampled either once or not at all, so that unsampled individuals can be accounted for in the transmission chains between sampled individuals. Thirdly, a complete transmission bottleneck meaning that only a single lineage is ever transmitted between hosts. In other words, the within-host coalescent process is bounded so that the most recent common ancestor within a host occurs after the date of infection (Carson et al. 2022).

The full bottleneck assumption can be problematic in settings where hosts are repeatedly sampled, as the resulting phylogenetic trees may have no compatible transmission trees (Romero-Severson et al. 2014, 2016). Therefore, we remove this complete bottleneck assumption, so that the phylogenetic trees are much more likely to have compatible transmission trees. Removing this assumption was needed to allow for multiple samples per host, but it is also important to note that a number of studies have found that the transmission bottleneck is only partial for many pathogens including HIV (Boeras et al. 2011), foot-and-mouth disease virus (Cortey et al. 2019), influenza (Ghafari et al. 2020), and *S. aureus* (Hall et al. 2019). Relaxing the transmission bottleneck assumption therefore leads to a more generally applicable model, in which it is possible to additionally estimate the scale of the transmission bottleneck.

We also relax the assumption of a constant within-host population size by allowing linear growth, following previous work on HIV (Romero-Severson et al. 2014, 2016; Leitner 2019). This linear growth model is a generalization of the constant population size model which can be obtained if the linear growth rate parameter is set to zero. It is also a generalization of a linear growth with complete transmission bottleneck model (Klinkenberg et al. 2017) since this can be obtained if the linear intersect is zero at the date of infection. The linear growth model therefore has several advantages, on top of being simple and statistically tractable, but other options such as an exponential or logistic growth model could also be used as will be discussed later.

Finally, in the transmission model, we add the possibility that hosts are sampled multiple times, while also retaining the possibility that some hosts are sampled only once or not at all. We make the specific choice that the transmission model up to the first sample for each host is exactly the same as previously formulated (Didelot et al. 2017). The times of any further sampling depend only on the first observation times, and not the infection times. Since the infection times and secondary observation times are conditionally independent given the primary observation times, we can infer the infection times without the need to formally define this aspect of the model. In the Methods section, we present a full mathematical description of this new extended model and show how Bayesian inference can be performed using a Markov chain Monte Carlo (MCMC) scheme with reversible jumps (Green 1995) to accommodate the nonconstant dimension of the parameter space.

## Results

### Exemplary Analysis of a Single Simulation

We simulate an outbreak with 100 observed hosts, each with 5 observations. The observation cutoff time *T* is determined by the simulation in order to return the correct number of observed hosts. The generation time and primary observation time are both Gamma distributed (see Epidemiological Model section) with shape and scale parameters equal to 2 and 1, respectively. Secondary observations are placed at intervals of 0.25 years following the primary observation. For the transmission model, the offspring distribution is negative binomial with mean equal to the basic reproduction number R=2, and the sampling proportion is π=0.8. The within-host pathogen population size is κ+λτ at time *τ* after infection, with κ=0.1 and λ=0.2. The resulting simulation contains 124 hosts, 4 of which are infected with 2 lineages at the time of infection, 1 with 3 lineages, and the remaining 119 with a single lineage.

We investigate the ability of our methodology to recover the model parameters used in the simulation, and to recover transmission links between individuals. We also investigate what benefits are obtained by including multiple observations per host. To this end, we construct additional phylogenetic trees by pruning the last observation for each host. Through repetition, we obtain phylogenetic trees with 4, 3, 2, and 1 observations per host under the same transmission network. By comparing inference outcomes from these 5 trees we can establish the extent to which estimates are improved through the inclusion of secondary observations.

We perform 12,000 MCMC iterations for each phylogenetic tree, using the first 2,000 as a burn-in. The prior distribution for *π* is uniform between 0 and 1, and the prior distributions for *R*, *κ*, and *λ* are exponential with mean 1. The posterior means and 95% credible intervals are shown in Table 1. These results demonstrate that we are able to recover the model parameters used in the simulation, even with no secondary observations. Comparing posterior estimates across the different trees indicates that our estimates of the transmission model parameters *R* and *π* are not considerably improved by the number of secondary observations. This makes sense, as most of the relevant information for these parameters is contained in the primary observation. However, the credible intervals for the coalescent model parameters *κ* and *λ* narrow as more secondary observations are added. Secondary observations provide considerable information about the within-host genomic diversity of infected hosts, leading to more precise estimates.

**Table 1.** Posterior estimates of the simulation study given as the posterior mean and 95% credible interval

	Observations per host				
	1	2	3	4	5
*π*	0.85[0.62,0.99]	0.83[0.62,0.99]	0.85[0.65,0.99]	0.83[0.63,0.99]	0.84[0.64,0.99]
*R*	2.32[1.84,2.83]	2.32[1.84,2.86]	2.27[1.78,2.80]	2.25[1.78,2.77]	2.25[1.79,2.78]
*κ*	0.18[0.01,0.38]	0.15[0.05,0.29]	0.10[0.03,0.19]	0.10[0.03,0.17]	0.11[0.05,0.17]
*λ*	0.19[0.01,0.58]	0.18[0.04,0.30]	0.23[0.14,0.33]	0.20[0.14,0.27]	0.21[0.15,0.27]

The model parameter is given in the left column, and the remaining columns indicate the number of observations per observed host. The values used in the simulation are π=0.8, R=2, κ=0.1, and λ=0.2.

**Fig. 1.**
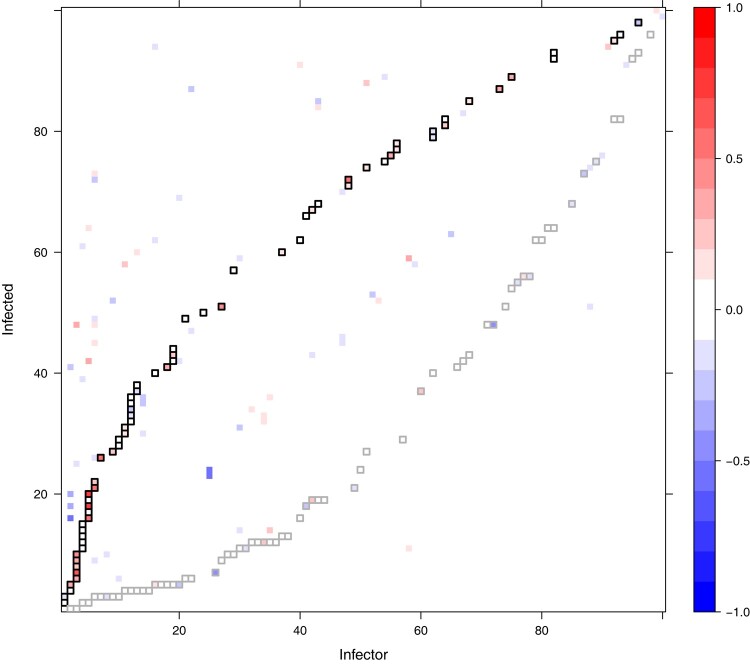
Difference in posterior probability estimates of transmission between a dataset with 1 observation per host and a dataset with 5 observations per host. The underlying transmission network remains the same; it is defined by the black squares, which show the true transmissions in the simulated dataset. The gray squares show the reverse relationship, switching the true infector and infected hosts. Black squares containing red demonstrate higher posterior probabilities being assigned to the true transmission links as a result of including more observations. Elsewhere, blue indicates lower posterior probabilities being assigned to incorrect transmission links.

**Fig. 2.**
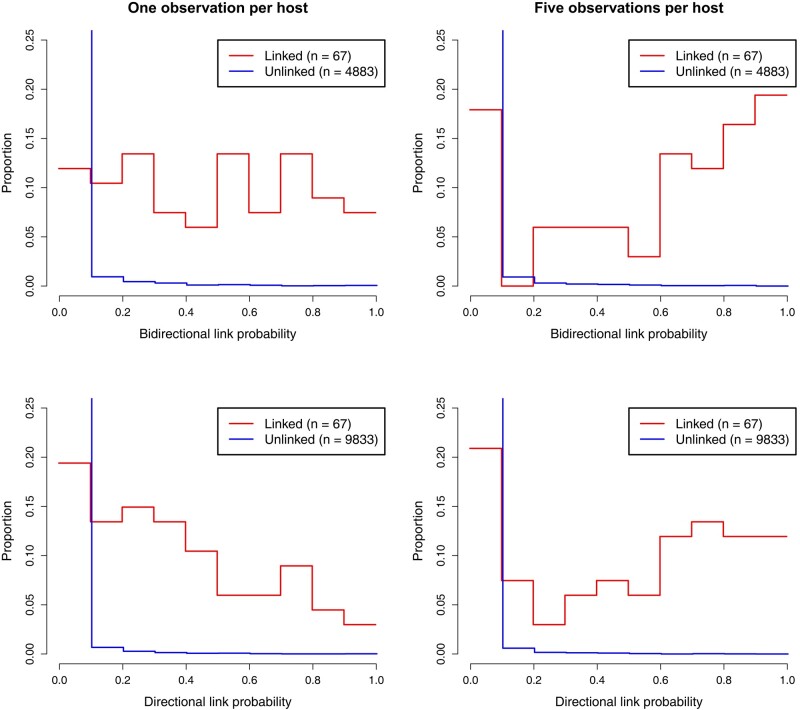
Distribution of posterior link probabilities inferred in the simulation studies with 1 (left) and 5 (right) observations per host. The top plots show bidirectional link probabilities in which the roles of infector and infected host may switch, the bottom plots show the directional link probabilities in which the infector and infected host must be correctly inferred. The red lines relate to pairs of individuals for which a transmission link exists, and the blue lines relate to pairs of individuals that are not linked.

In order to evaluate our ability to reconstruct transmission links, we look at transmissions between observed hosts. Out of the 100 observed hosts, 67 are infected by another sampled individual. From our estimated transmission trees, we consider both directional transmission links, where we must correctly establish the infector and infected host, and bidirectional transmission links, where a transmission link is established but the roles of infector and infected may swap. We define 0.5 as the posterior probability threshold for a transmission being identified, and define the sensitivity as the proportion of correctly identified transmission links (true positive rate). For the phylogenetic tree with 1 observation per host, we obtain a sensitivity of 0.51 for bidirectional transmission links, and 0.28 for directional transmission links (supplementary fig. S1, Supplementary Material online). For the phylogenetic tree with 5 observations per host, the sensitivity increases to 0.64 for bidirectional transmission links and 0.55 for directional transmission links (Fig. 1). The specificity (true negative rate) is greater than 0.996 in all cases. The full distributions of posterior probability estimates in each setting are shown in Fig. 2. Increasing the number of secondary observations allows us to better reconstruct transmission links, and crucially, to better distinguish the direction of transmission.

The within-host population model plays a key role in our ability to establish transmission links. If the transmission of multiple lineages is more common, the posterior probabilities of transmission links will tend to be lower. For example, repeating the simulation process above with a full bottleneck (fixing κ=0) results in a bidirectional (directional) sensitivity of 0.57 (0.43) with 1 observation per host, and 0.75 (0.63) with 5 observations per host, all higher than in the previous results with a partial bottleneck. On the other hand, increasing to κ=0.4 leads to a bidirectional (directional) sensitivity of 0.34 (0.25) with 1 observation per host, and 0.54 (0.39) with 5 observations per host, all lower than the example with κ=0.1.

When only a single genome per host is used, we are able to run the original TransPhylo algorithm (Didelot et al. 2017) for comparison. The estimate of *π* is 0.93 with credible interval [0.76,1.00], and the estimate of *R* is 2.38 with credible interval [1.88,2.95], which are similar to the estimates obtained previously with 1 observation per host (Table 1). The probabilities for who infected whom are shown in supplementary fig. S2, Supplementary Material online. The bidirectional (directional) sensitivity is 0.61 (0.37), as illustrated in supplementary fig. S3, Supplementary Material online. Since a small value of κ=0.1 is used in the simulation, the strict bottleneck assumption in TransPhylo is advantageous here, whereas using a relaxed bottleneck leads to additional uncertainty on who infected whom. TransPhylo would perform comparatively less well if the true bottleneck was more relaxed.

### Benchmarking Using Multiple Simulations

We now repeat this process, again using a simulated dataset with 100 hosts and 5 observations per host; but performing the inference on simulations generated from a range of key parameters (*π*, *R*, *λ*, and *κ*), totalling 43 datasets. As previously, both the generation time distribution and primary observation time distribution follow a Gamma distribution with shape parameter 2 and scale parameter 1, and secondary observations occur 0.25 years later than the previous sample.

**Fig. 3.**
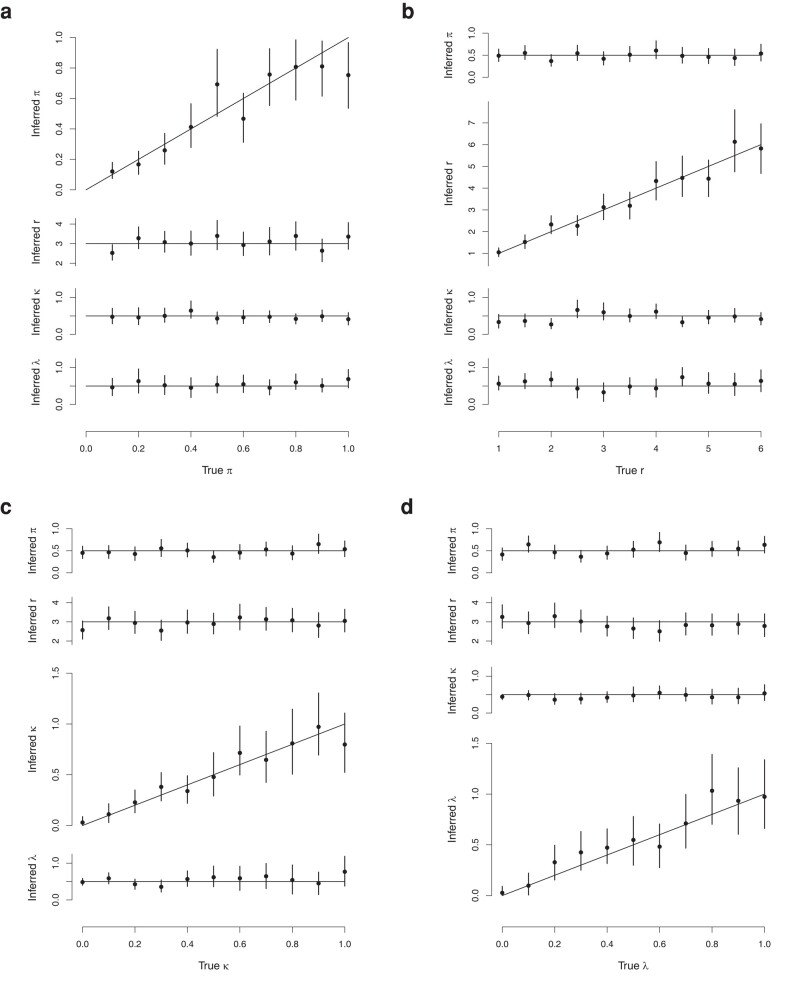
Varying the 4 key simulation parameters. Vertical bars show 95% central credible intervals, while solid circles show posterior means. Horizontal or diagonal lines show true values for simulations. a) Varying *π*. b) Varying *R*. c) Varying *κ*. d) Varying *λ*.

For the MCMC chains, we obtain 12,000 samples, and discard the first 2,000 as a burn-in. Figure 3 shows the posterior parameter estimates. The vertical lines show central 95% credible intervals for each parameter, and the posterior mean is shown with a solid circle. The horizontal and diagonal lines indicate the true parameter values used to generate the data. These results demonstrate strong performance of the algorithm across very different simulation settings.

The linear growth assumption of the within-host population size model is unlikely to resemble a real-world population, and so we also test for robustness to the misspecification of the within-host population model. We repeat the inference, but fix the within-host population growth rate *λ* at either half or double the true value. The posterior estimates are shown in supplementary fig. S4, Supplementary Material online. Most notably, the misspecification biases our estimates of the initial pathogen population size *κ*. There is a strong negative correlation between *λ* and *κ*, so that when *λ* is set lower (higher) *κ* is overestimated (underestimated). There are smaller changes in the transmission model parameters, with a lower *λ* resulting in higher estimates of *π* and lower estimates of *r*, but the true values for these parameters usually remain within the 95% credible intervals. These results suggest that estimates of the transmission model parameters are reasonably robust to the misspecification of the within-host population model. However, caution is warranted when interpreting the estimates of the within-host model parameters. We can reasonably conclude, for instance, that different estimates of the initial population size *κ* may be obtained under different growth models.

### Application to *Pseudomonas aeruginosa* Transmission Between Cystic Fibrosis Patients

We reanalyzed previously published genomic data from Danish cystic fibrosis (CF) patients infected with *P. aeruginosa* (Marvig et al. 2013). This dataset included 42 genomes from 14 patients, sampled over almost 40 years between 1972 and 2008, after exclusion of hypermutator and recombinant isolates (Marvig et al. 2013). Previous studies explored within-host evolutionary dynamics (Yang et al. 2011), variations in gene content (Rau et al. 2012) and comparative adaptation in CF human hosts (Marvig et al. 2013). The hosts are designated CFXXX as in these previous studies. We use as our starting point the dated phylogeny previously computed (Marvig et al. 2013) using BEAST (Suchard et al. 2018) and shown in supplementary fig. S5, Supplementary Material online. It was previously noted (Yang et al. 2011) that one of the individuals (CF66) had been infected twice in the 1970s and the 1990s, and so we modeled this as 2 separate hosts (labeled CF66a and CF66b). Infection with *P. aeruginosa* can be stable over long periods of time in CF patients (Rossi et al. 2021) and indeed some of the patients had been sampled, and found positive, over a period of more than 20 years (Marvig et al. 2013). We therefore set the generation time distribution to be Gamma with shape 2 and scale 5, resulting in a mean of 10 years, standard deviation of 7 years, and 95% range of 1.2 to 27.9 years. The last samples were from 2008 and the exact end of the sampling period was unclear from previous publications but we set it to the end of 2009.

**Fig. 4.**
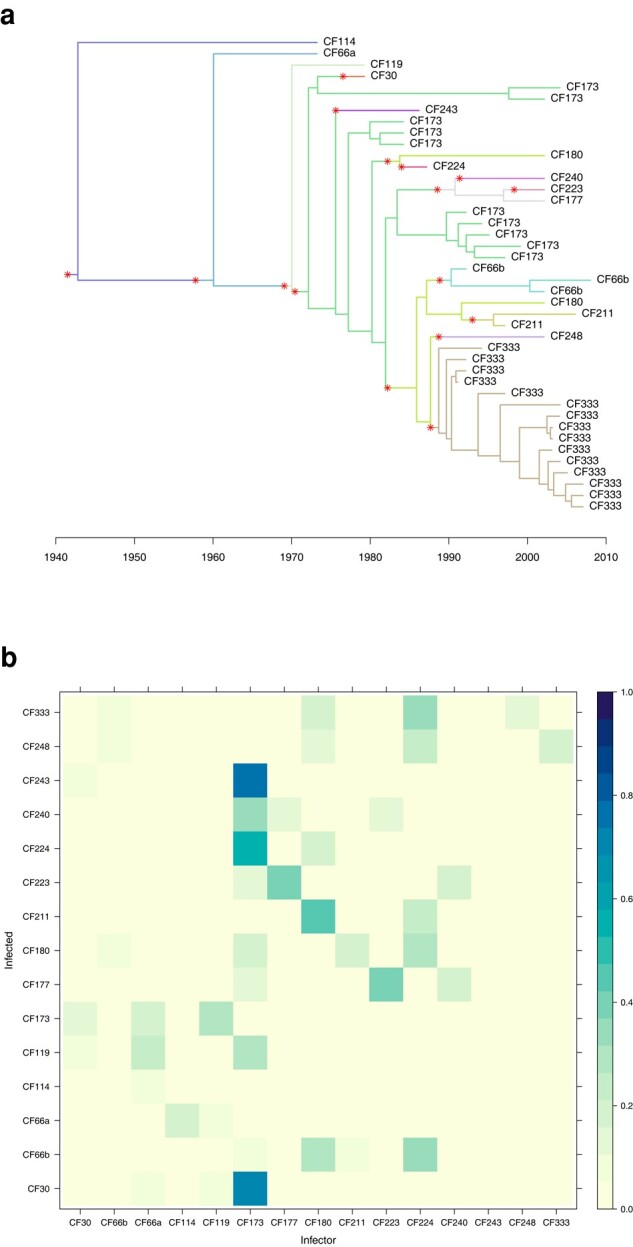
Transmission analysis of *P. aeruginosa*. a) Dated phylogeny colored by host according to the iteration with highest posterior probability. b) Matrix of transmission probabilities from each host (row) to any other (column).

We performed 4 separate runs of 100,000 iterations, which took approximately 3 h on a standard laptop computer. For each of the 4 parameters *π*, *R*, *κ* and *λ* we checked that the effective sample size in each run was over 1,000 and the multivariate Gelman-Rubin statistic comparing runs was less than 1.1 (Brooks and Gelman 1998). Figure 4a shows the dated tree, colored by host according to the MCMC iteration with the highest posterior probability. Changes in colors along the branches of the tree correspond to transmission events and are highlighted with red stars. Note that there are 2 simultaneous stars leading to the 2 genomes from patient CF180. These both correspond to infection from CF173, with the 2 lineages being transmitted through the relaxed transmission bottleneck. Figure 4a is useful to illustrate the coloring process which relates the phylogenetic tree to the transmission tree. However, this only represents a single transmission configuration explored by the MCMC, and other iterations of the MCMC would look different, maybe with some of the same transmission events and others being different. It is therefore important to consider the probability of the transmission events. Figure 4b shows the matrix of probabilities of infection from each host to another, computed as the frequency of each transmission event across all MCMC iterations.

Supplementary figure S6, Supplementary Material online shows the trace and density of the parameters estimated in a single MCMC run. The sampling proportion was estimated to be π=0.65, with a wide 95% credible interval [0.30 to 0.96]. The reproduction number was R=1.20[0.58to1.99]; as the credible interval includes 1, it is not clear if the outbreak has the potential to cause a self-sustained epidemic. The within-host linear growth rate was λ=0.56[0.16to1.09] per year, which is lower than the prior exponential with mean 1. On the other hand, the within-host starting population size was κ=2.16[0.41to5.05] which is higher than the prior exponential with mean 1. This suggest that the bottleneck was not complete, and indeed attempting to fit the model with κ=0 is impossible as it leads to a likelihood of zero. This is caused by the 2 samples from CF180 and the 10 samples from CF173 being “inconsistent” as previously designated for samples from 2 hosts that cannot be explained by transmission of a single lineage (Romero-Severson et al. 2014, 2016). The individual CF173 was found to have infected at least 3 other hosts (CF30, CF224, and CF243) with probability higher than 50% (Fig. 4b). These transmission events and their directionality are made clear by the paraphyletic relationship of the 10 samples from CF173 as shown in Fig. 4a (Leitner 2019). In contrast, the 15 samples from CF333 formed a single monophyletic clade (Fig. 4a) so that they are unlikely to have infected many others except maybe CF248 (Fig. 4b).

### Application to a Nosocomial Outbreak of *Klebsiella pneumoniae*

An outbreak of carbapenem-resistant *K. pneumoniae* expressing the *bla*${}_{\mathrm{OXA}-232}$ gene was identified over the course of 40 weeks at a single healthcare institution in California (Yang et al. 2017). A total of 17 infected patients were identified, from which 32 isolates were taken between 2014 October 12 and 2015 July 17. Case finding was performed using all samples in the 2014 and 2015 calendar years (Yang et al. 2017) and so we set the date for the end of the sampling period to the end of 2015. Whole-genome sequencing was applied to these *K. pneumoniae* isolates and a dated phylogeny was computed previously (Yang et al. 2017) using BEAST (Suchard et al. 2018) which is shown in supplementary fig. S7, Supplementary Material online. The hosts are labeled either PtXXX if they were symptomatic or CPtXXX if they were colonized, as in the previous study (Yang et al. 2017). We set the generation time distribution to be exponential with mean 0.5 year, following a previous study of another *K. pneumoniae* hospital outbreak (van Dorp et al. 2019). This diffuse distribution is well suited to capture transmission via hospital equipment contamination as was previously suggested (Yang et al. 2017). We used the same number of MCMC runs, length of runs, and convergence diagnostics as in the previous application.

Supplementary figure S8, Supplementary Material online shows the trace and density of the parameters estimated in a single MCMC run. The sampling proportion was estimated to be high, with π=0.88[0.60to0.99], suggesting that there were only few missing transmission links between the 17 sampled patients. The basic reproduction number was R=0.97[0.37to1.74], with the credible interval including the value of 1 needed for an outbreak to spread beyond a few cases. The within-host linear growth rate was λ=0.49[0.03to1.28] per year and the within-host population size at time of infection was κ=0.066[0.009to0.158]. This is lower that the prior exponential with mean 1 and suggests that the transmission bottleneck was almost complete during this small outbreak. However, the transmission bottleneck was not absolutely complete, as indicated by the fact that fitting our model with κ=0 would result in a likelihood equal to zero. This is because the 6 samples from Pt6 and the 2 samples from Pt9 are inconsistent, as can be seen in the dated phylogeny on supplementary fig. S7, Supplementary Material online.

**Fig. 5.**
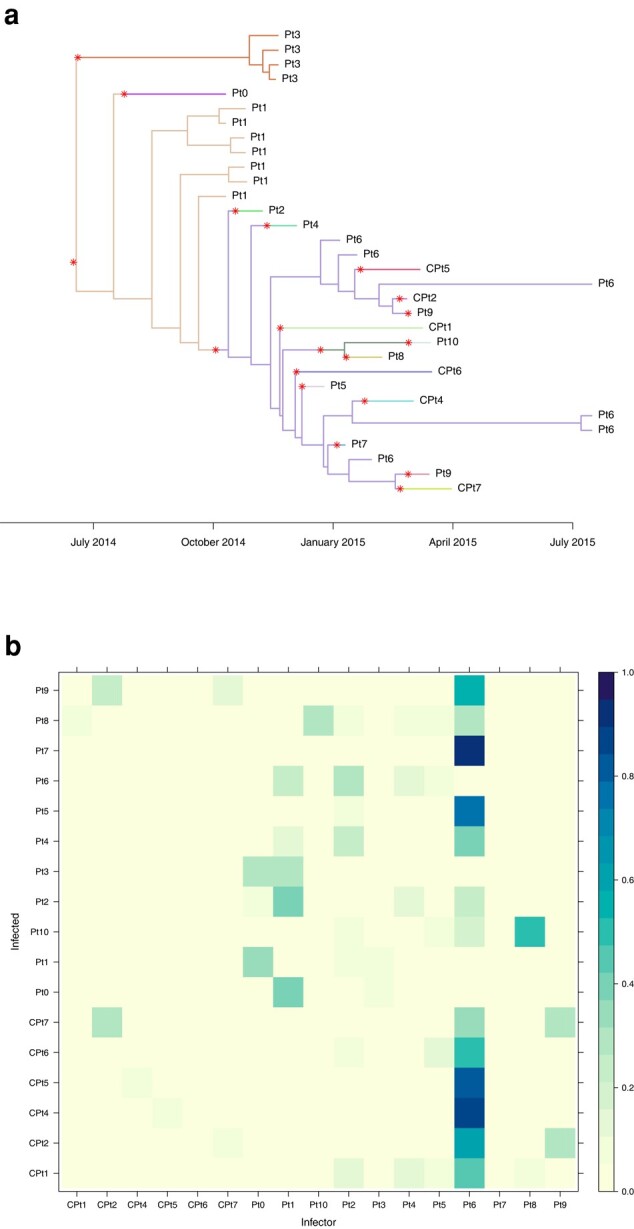
Transmission analysis of *K. pneumoniae*. a) Dated phylogeny colored by host according to the iteration with highest posterior probability. b) Matrix of transmission probabilities from each host (row) to any other (column).

Figure 5a shows the dated tree colored by host according to the MCMC iteration with highest posterior probability, while Fig. 5b shows the posterior probabilities of infection from any host to any other. For example, a high probability of transmission was found from Pt8 to Pt10, which is consistent with the fact that these 2 patients were staying in neighboring rooms for 2 weeks (Yang et al. 2017). Strikingly, according to our analysis, patient Pt6 had a greater than 50% posterior probability of having infected 7 other patients (CPt2, CPt4, CPt5, CPt6, Pt5, Pt7, and Pt9). There were 6 genomes isolated from Pt6, with dates ranging from 2015 January 7 to 2015 July 17 which is more than half of the overall sampling period. The specimen types for these isolates were quite diverse: 3 from blood, 1 rectal, and 2 from bile (Yang et al. 2017), suggesting that the patient was infected long enough for the pathogen to spread throughout their body. While other patients in the study do present a similar number of samples, a comparable variety of originating tissues, and a similarly long infection duration—for instance patient Pt1, with 7 genomes from respiratory, abdominal, and blood specimen over a period of several months—that does not translate in a similar amount of infection events estimated by our method. In fact, the genetic diversity of isolates from Pt6 appears to be very high (Fig. 5a), thus backing our inference that Pt6 is a superspreading individual (Lloyd-Smith et al. 2005). This could not have been detected without the use of multiple genomes.

## Discussion

We have described new methodology for inferring who infected whom from a dated phylogenetic tree in which hosts have potentially been sampled multiple times. A key change compared to previous work (Didelot et al. 2014, 2017) is the removal of the full transmission bottleneck, meaning that hosts may be infected with multiple lineages from the transmission donor. Without this change many phylogenetic trees with multiple samples per host would not support compatible transmission trees (Romero-Severson et al. 2014, 2016; Leitner 2019). Indeed the 2 real datasets we analyzed, corresponding to outbreaks of *P. aeruginosa* and *K. pneumoniae*, could not be explained without relaxing the transmission bottleneck. Most previous transmission analysis methods could not accommodate more than a single genome per host, so that leaves would need to be pruned from the phylogenetic tree in order to undertake transmission inference (Xu et al. 2020), leading to less informative outcomes. Under our new methodology, we are able to incorporate multiple samples per host, resulting in the stronger identification of transmission links and their direction, as was showed when analyzing simulated datasets.

We build upon previous work (Didelot et al. 2014, 2016) that performs transmission analysis by coloring the branches of a preestablished dated phylogeny. This allows us to model the relationship between transmission tree and phylogeny through an explicit within-host evolutionary model, to develop an explicit transmission model in which sampled and unsampled individuals are featured, and to achieve better scalability by separating phylogenetic inference from its epidemiological interpretation. On the other hand, relying on a fixed dated tree could be problematic as this does not account for the uncertainty in the phylogeny or the dates of common ancestors. When this uncertainty is captured using a Bayesian phylogenetic method (Didelot et al. 2018; Suchard et al. 2018; Bouckaert et al. 2019), this effect can be tested by applying analysis to multiple samples instead of a single fixed tree (Nylander et al. 2008). However, this was found in practice to make little difference to the inferred transmission probabilities and parameters (Didelot and Parkhill 2022).

Our method implements a general pathogen population growth model rather than using the constant bounded coalescent model, in which the population size is constant and the most recent common ancestor is forced to occur after the infection time (Carson et al. 2022). By removing this restriction, we were able to model transmission through a relaxed bottleneck. The main restriction on the choice of model is that we must be able to calculate the likelihood of the phylogenetic tree, which in turn means that the coalescence rate must be integrable. However, this is not a strong requirement, as many widely used models satisfy it—among them the exponential growth model, the logistic growth model, or any piecewise models with separate growth and decay phases. For the work presented here, we used a linear growth model, which has been used before in HIV work (Romero-Severson et al. 2014, 2016; Leitner 2019), but for most other pathogens there is little information about which within-host population size model is most realistic (Didelot et al. 2016). We demonstrated that using phylogenetic trees with multiple samples per host improves the estimation of the population model parameters. With sufficient samples per host it should be possible to determine which within-host population size models are more strongly supported by the data, for example and comparing the evidence of each model (Friel and Wyse 2012).

Our methodology maintains some of the assumptions from previous work (Didelot et al. 2017), for example the sampling proportion and reproduction number are assumed to remain constant through time. In many settings, users would have knowledge about whether and how the sampling proportion varied over time, for example by looking at the number cases for which genomic sequences are available divided by the number of confirmed cases (Jelley et al. 2022). This information could be integrated relatively easily into an analysis, by having users supply a function π(t) instead of the constant *π*. On the other hand, it would often be interesting to infer variations in the reproduction number R(t), since this would provide an additional genomic-based estimate compared to existing methods based on incidence data (Wallinga and Teunis 2004; Cori et al. 2013). A simple approach would be to use a stepwise constant function. The dates of these steps may be fixed based on real-world policy changes, such as intensifying monitoring in response to an outbreak, or potentially inferred via change point detection (Tartakovsky and Moustakides 2010).

In conclusion, we presented a new Bayesian inference method for the reconstruction of transmission trees from dated phylogenetic trees in which hosts are sampled multiple times. This method is implemented in a R package that extends TransPhylo and is available at https://github.com/DrJCarson/TransPhyloMulti. When applied to multiple sampled genomes from several infected individuals, our method has the potential to improve our understanding of both the within-host and between-host dynamics of many pathogens causing infectious disease.

## Materials and Methods

### Notation

Let us denote P as the dated phylogenetic tree, T as a transmission tree, $\theta_{P}$ as the coalescent model parameters, and $\theta_{T}$ as the transmission model parameters. We want to sample from the posterior distribution


(1) $$p(\theta_{P},\theta_{T},T|P)\propto p(P|T,\theta_{P})p(T|\theta_{T})p(\theta_{T})p(\theta_{P}),$$

where the term $p(P|T,\theta_{P})$ is the likelihood of the coalescent model conditional on a given transmission tree, the term $p(T|\theta_{T})$ is the likelihood of the transmission model, and the terms $p(\theta_{P})$ and $p(\theta_{T})$ are prior distributions.

We parameterize the transmission tree T as follows. Let *x* be a vector of infection times such that element $x^{j}$ gives the infection time of host *j*. Likewise let *A* be a vector of infectors, so that if $A^{j}=i$ then host *j* was infected by host *i*. We indicate the root host by setting $A^{j}=0$. Primary observation times are denoted by vector *y*, with the corresponding host denoted by vector $H_{y}$. Secondary observation times are denoted by vector *z*, with host $H_{z}$.

For the phylogenetic tree P, we need to consider the leaf and coalescent times. The leaves correspond to observations under the transmission tree. We denote the vector of leaf times *s* and corresponding hosts $H_{s}$, noting that s=(y,z) and that $H_{s}=(H_{y},H_{z})$. We indicate the parent node of each sample using vector $C_{s}$. The coalescent node times are denoted by vector *u*, and their parent nodes $C_{u}$. We again denote the root node with $C_{u}^{j}=0$.

**Fig. 6.**
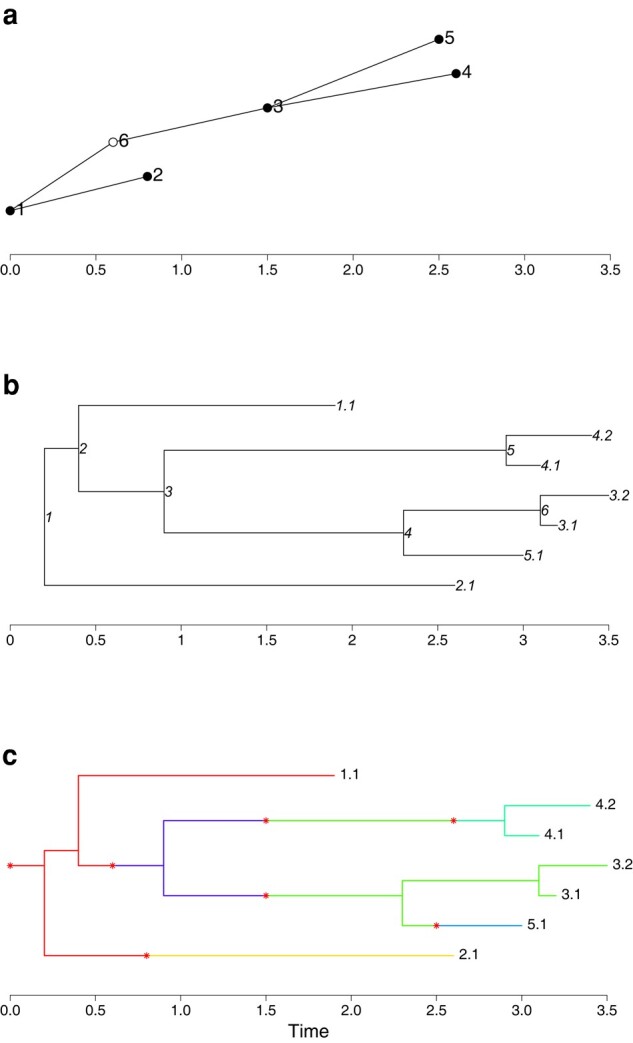
a) Example transmission tree with 6 hosts. Points indicate the infected times of each host. Filled circles show observed hosts, and empty circles show unobserved hosts. b) Example phylogenetic tree with 7 leaves from 5 observed hosts. Leaf labels indicate the host, followed by the sample number for that host. Each coalescence node is given a label. c) Example colored phylogenetic host with 7 leaves from 5 observed hosts, and 6 hosts overall. The branch color indicates the host, and the asterisks indicate transmissions. Here, host 3 is infected with 2 lineages.

Figure 6a demonstrates a transmission tree with


$$x=\left(\begin{array}{c} 0.0\\ 0.8\\ 1.5\\ 2.6\\ 2.5\\ 0.6 \end{array}\right),\;A=\left(\begin{array}{c} 0\\ 1\\ 6\\ 3\\ 3\\ 1 \end{array}\right).$$

That is, host 1 infects hosts 2 and 6, host 6 infects host 3, and host 3 infects hosts 4 and 5. In addition, we have primary and secondary observations (not shown), for example


$$y=\left(\begin{array}{c} 1.9\\ 2.6\\ 3.2\\ 3.1\\ 3.0 \end{array}\right),\;H_{y}=\left(\begin{array}{c} 1\\ 2\\ 3\\ 4\\ 5 \end{array}\right),\;z=\left(\begin{array}{c} 3.5\\ 3.4 \end{array}\right),\;H_{z}=\left(\begin{array}{c} 3\\ 4 \end{array}\right)$$

indicates that hosts 1, 2, and 5 are observed once, hosts 3 and 4 are observed twice, and host 6 is unobserved.

Figure 6b shows an example phylogenetic tree obtained by combining the primary and secondary observations from the transmission tree. Here,


$$s=\left(\begin{array}{c} 1.9\\ 2.6\\ 3.2\\ 3.1\\ 3.0\\ 3.5\\ 3.4 \end{array}\right),\;u=\left(\begin{array}{c} 0.2\\ 0.4\\ 0.9\\ 2.3\\ 2.9\\ 3.1 \end{array}\right),\;H_{s}=\left(\begin{array}{c} 1\\ 2\\ 3\\ 4\\ 5\\ 3\\ 4 \end{array}\right),\;C_{s}=\left(\begin{array}{c} 2\\ 1\\ 6\\ 5\\ 4\\ 6\\ 5 \end{array}\right)\;C_{u}=\left(\begin{array}{c} 0\\ 1\\ 2\\ 3\\ 3\\ 4 \end{array}\right).$$

We can represent both the transmission and phylogenetic trees as a colored phylogenetic tree, as shown in Fig. 6c. Doing so highlights that each coalescent event is now assigned to a host.

### Epidemiological Model

The epidemiological model is a stochastic branching process in which infected individuals transmit to secondary cases (offspring). The number of offspring *k* is sampled from the offspring distribution α(k), assumed to be a negative binomial distribution with parameters (r,p), i.e.


(2) α(k)=(k+r−1k)pk(1−p)r.

The time between the primary and any secondary infection is sampled from the generation time distribution γ(τ), which typically follows a Gamma distribution with known parameters.

Under a *finished outbreak* scenario, each host is assumed to be observed with probability *π*. The time between the host being infected and first being observed is sampled from the observation time distribution σ(τ). As with the generation time distribution, this is typically a Gamma distribution with known parameters.

In some applications, observations occur over a restricted time interval, or possibly set of time intervals. In such applications, the probability of a host being observed depends on their infection time. An example, we will look at is the *ongoing outbreak* scenario, in which there is an observation cutoff time *T*. In this scenario, a host infected at time *t* is observed with probability


$$\zeta (t)=\pi \int_{0}^{T-t}\sigma (\tau )\;d\tau .$$

In other words, we use the same observation distribution as the finished outbreak scenario, but treat observations later than *T* as censored.

Finally, hosts may be observed multiple times. We assume that any host can only be infected once, and that any subsequent observations relate to the same infected period. We define β(b) as the distribution for the number of secondary observations b≥0, and $\rho (\tau_{1\;:\;b})$ as the distribution for the times between the secondary observations and the primary observation assuming that b≥1. Note that it is possible for the time between observations to be zero, meaning that multiple observations occur at the primary observation time.

Secondary observations are an additional modeling component to the previous version of TransPhylo (Didelot et al. 2017). However, by assuming that the secondary observation times depend only on the primary observation times, we can undertake inference in a similar manner without formally specifying these distributions. Under our modeling assumptions we can express the likelihood of the transmission tree as


(3) $$\begin{array}{rl} \;p(T|\theta_{T}) & =p(x,y,z,A,H_{y},H_{z}|\theta_{T})\\ & =p(z,H_{z}|y,H_{y})p(y,H_{y}|x,A,\theta_{T})p(x,A|\theta_{T}), \end{array}$$

where *x*, *A*, and $\theta_{T}$ are parameters we are trying to estimate, and *y*, *z*, $H_{y}$, and $H_{z}$ are fixed by the dated phylogenetic tree. Within a Metropolis–Hastings algorithm, when we propose new values $x^{\prime }$ and $A^{\prime }$ (giving a new transmission tree $T^{\prime }$) or $\theta_{T}^{\prime }$, the term $p(z,H_{z}|y,H_{y})$ will cancel in the likelihood ratio, i.e.


(4) $$\frac{\;p(T|\theta_{T})}{p(T^{\prime }|\theta_{T}^{\prime })}=\frac{\;p(y,H_{y}|x,A,\theta_{T})p(x,A|\theta_{T})}{p(y,H_{y}|x^{\prime },A^{\prime },\theta_{T}^{\prime })p(x^{\prime },A^{\prime }|\theta_{T}^{\prime })}.$$

Consequently, $p(z,H_{z}|y,H_{y})$ does not need to be explicitly calculated to determine if proposals are accepted or rejected, and practically can be excluded from the transmission tree likelihood altogether.

#### Host Inclusion and Exclusion

Our goal is to infer a transmission tree from a dated phylogenetic tree. This can be visualized as *coloring* the branches of the phylogenetic tree, where each color represents a distinct host. For a host to appear on the phylogenetic tree they must either be observed directly or be an ancestor to a different observed host. We refer to such hosts as *included* hosts. In many applications, the number included hosts is dwarfed by the number of hosts implied by the epidemiological model to not appear on the phylogenetic tree (*excluded* hosts). Examples include when *π* is small, or when *r* is large in an ongoing outbreak scenario. In the latter case, a large number of hosts will be infected shortly before the observation cutoff time, and so will be excluded with high probability. For this reason, we instead formalize a transmission model for only the included hosts.

Define ω(t) as the exclusion probability of a host infected at time *t*. Assuming that *T* is the cutoff time for observations ω(t)=1 for t≥T. We can then define the following recursive relationships.

The exclusion probability of an offspring from a host infected at time *t* is


(5) $$\bar{\omega }(t)\;=\int_{0}^{\infty }\omega (t+\tau )\gamma (\tau )\;d\tau .$$

The probability that all offspring from an individual infected at time *t* are excluded is


(6) $$\phi (t)=\sum_{k=0}^{\infty }\alpha (k)\bar{\omega }{\left(t\right)}^{k}.$$

The exclusion probability of an individual infected at time *t* is


(7) $$\begin{array}{rl} \omega (t) & =(1-\zeta (t))\phi (t)\\ & =(1-\zeta (t))\sum_{k=0}^{\infty }\alpha (k){\left(\int_{0}^{\infty }\omega (t+\tau )\gamma (\tau )\;d\tau \right)}^{k}. \end{array}$$

That is, the probability of the host being unobserved and having no included offspring. In the finished outbreak scenario, the recursive relationship is simply


(8) $$\omega_{*}=(1-\pi )\sum_{k=0}^{\infty }\alpha (k)\omega_{*}^{k},$$

with $\omega_{*}$ being the exclusion probability for every host. Note that these calculations do not depend on the secondary observation times or their distribution.

#### Numerical Approximations

The exclusion probabilities are intractable, and so we use numerical approximations. For example, consider the ongoing outbreak scenario with observation cutoff time *T*. For t≥T, $\omega_{t}=1$, and so


(9) $$\bar{\omega }(t)\;=\int_{t}^{T}\gamma (\tau -t)\omega (\tau )\;d\tau \;+\int_{T}^{\infty }\gamma (\tau -t)\;d\tau .$$

The second term can be computed explicitly, and the first term can be approximated using the trapezoid method:


(10) $$\int_{t}^{T}\gamma (\tau -t)\omega (\tau )\;d\tau \approx \sum_{i=0}^{k}c_{i}\gamma ((k-i)\Delta t)\omega (t_{i})\Delta t,$$

where $c_{i}=1$ for 0<i<k and $c_{i}=0.5$ otherwise, and $t_{i}=T-i\Delta t$. Assuming γ(0)=0:


(11) $$\bar{\omega }(t)\approx F(t)+\sum_{i=0}^{k-1}c_{i}\gamma ((k-i)\Delta t)\omega (t_{i})\Delta t,$$

where $F(t)\;=\int_{T}^{\infty }\gamma (\tau -t)\;d\tau$.

Using the probability generating function of a negative binomial distribution with parameters *r* and *p*, we can evaluate


(12) $$\phi (t)={\left(\frac{\;p}{1-(1-p)\bar{\omega }(t)}\right)}^{r},$$

and finally


(13) ω(t)=(1−ζ(t))ϕ(t).

Both will be approximate owing to the approximation of $\bar{\omega }(t)$. All 3 exclusion probabilities are therefore approximated by iterating backwards through time from *T* in discrete steps of size Δt.

#### Transmission Tree Likelihood

We can now define a likelihood for the transmission tree for only included individuals. Throughout we will set *T* as the cutoff time for observations. Consider first the root host (the first infected individual in our transmission chain) with infection time $x^{1}$, and let $I^{1}=1$ denote that the root host is included. The probability that the root host is unobserved (denoted by $S^{1}=0$) given that they are included is


(14) $$\begin{array}{rl} \;p(S^{1}=0|I^{1}=1,x^{1}) & =\frac{\;p(I^{1}=1|S^{1}=0,x^{1})p(S^{1}=0|x^{1})}{p(I^{1}=1|x^{1})}\\ & =\frac{\left(1-\phi (x^{1}))(1-\zeta (x^{1})\right)}{1-\omega (x^{1})}, \end{array}$$

and the probability that the root host is observed ($S^{1}=1$) is


(15) $$\begin{array}{rl} \;p(S^{1}=1|I^{1}=1,x^{1}) & =\frac{\;p(I^{1}=1|S^{1}=1,x^{1})p(S^{1}=1|x^{1})}{p(I^{1}=1|x^{1})}\\ & =\frac{\zeta (x^{1})}{1-\omega (x^{1})}. \end{array}$$

In the event the root host is observed, we also need to calculate the density of the primary observation time $y^{1}$,


(16) $$p(y^{1}|S^{1}=1,x^{1})=\frac{\sigma (y^{1}-x^{1})}{\int_{0}^{T-x^{1}}\sigma (\tau )\;d\tau },\;x^{1}<y^{1}<T.$$

Additionally the full transmission tree likelihood incorporates the density of the secondary observation times. However, when it comes to undertaking inference these terms will cancel out, and so we skip this step.

Second, we calculate the probability that the root host has $d^{1}$ included offspring. The probability of a host infected at time *t* producing *d* included offspring is


(17) p(d∣t)=∑k=d∞α(k)p(d∣k,t)=∑k=d∞α(k)(kd)ω¯(t)k−d(1−ω¯(t))d.

We then need to condition on whether or not the root host was sampled. If the root host was not sampled, they must produce at least 1 included offspring to be included, and so


(18) $$\begin{array}{rl} & \;p(d^{1}|I^{1}=1,S^{1}=0,x^{1})\\ & =\frac{\;p(I^{1}=1|d^{1},S^{1}=0,x^{1})p(d^{1}|S^{1}=0,x^{1})}{p(I^{1}=1|S^{1}=0,x^{1})}\\ & =\frac{\;p(d^{1}|x^{1})}{1-\phi (x^{1})},\;d^{1}>0. \end{array}$$

If the root host was sampled, then it is included for any value of $d^{1}$, and so


(19) $$\begin{array}{rl} & \;p(d^{1}|I^{1}=1,S^{1}=1,x^{1})\\ & =\frac{\;p(I^{1}=1|d^{1},S^{1}=1,x^{1})p(d^{1}|S^{1}=1,x^{1})}{p(I^{1}=1|S^{1}=1,x^{1})}\\ & =p(d^{1}|x^{1}),\;d^{1}\geq 0. \end{array}$$

In the event $d^{1}>0$, we also calculate the density of the transmission times for any included offspring. Denoting $H^{1}$ as the offspring labels, ${\bar{x}}^{1}=\{x^{j}|j\in H^{1}\}$ as the set of offspring infection times, and ${\bar{I}}^{1}=1$ that the set of offspring are included, the likelihood contribution is


(20) $$\begin{array}{rl} \;p({\bar{x}}^{1}|{\bar{I}}^{1}=1,x^{1}) & =d^{1}!\prod_{\;j\in H^{1}}\frac{\;p(I^{j}=1|x^{j})p(x^{j}|x^{1})}{p(I^{j}=1|x^{1})}\\ & =d^{1}!\prod_{\;j\in H^{1}}\frac{\left(1-\omega (x^{j}))\gamma (x^{j}-x^{1}\right)}{1-\bar{\omega }(x^{1})}. \end{array}$$

The $d^{1}!$ term arises from the fact that the infection times are labeled according to host, and the host labels are arbitrary. If we imagine simulating a transmission tree, the offspring infection times can be generated in any order (of which there are $d^{1}!$ possible orderings) to produce the same transmission tree.

In summation, the likelihood contribution (sans secondary observations) for the root host in the unobserved case is


(21) LT1(θT)=(1−ϕ(x1))(1−ζ(x1))1−ω(x1)×11−ϕ(x1)∑k=d1∞α(k)(kd1)ω¯(x1)k−d1(1−ω¯(x1))d1×d1!∏j∈H1(1−ω(xj))γ(xj−x1)1−ω¯(x1)=(1−ζ(x1))1−ω(x1)∑k=d1∞α(k)(kd1)ω¯(x1)k−d1d1!∏j∈H1(1−ω(xj))γ(xj−x1),

and for the observed case is


(22) LT1(θT)=ζ(x1)1−ω(x1)σ(y1−x1)∫0T−x1σ(τ)dτ×∑k=d1∞α(k)(kd1)ω¯(x1)k−d1(1−ω¯(x1))d1×d1!∏j∈H1(1−ω(xj))γ(xj−x1)1−ω¯(x1)=πσ(y1−x1)1−ω(x1)∑k=d1∞α(k)(kd1)ω¯(x1)k−d1d1!∏j∈H1(1−ω(xj))γ(xj−x1).

The full likelihood is calculated by recursion, applying the same density calculations to each included host, i.e.


(23) $$p(T|\theta_{T})=\prod_{\;j=1}^{N}L_{T}^{j}(\theta_{T}),$$

with *N* being the total number of included hosts. Note that in doing so, with the exception of the root host, the terms $1-\omega (x^{j})$ will cancel in the likelihood.

Methods for simulating transmission trees are provided in supplementary text S1, Supplementary Material online.

### Coalescent Model

In the original version of TransPhylo the coalescent model used was the bounded coalescent (Carson et al. 2022). This model follows the standard coalescent model with heterochronous sampling (Drummond et al. 2002), but conditions all lineages to coalesce before the infection time of each host. Here, we need to choose a coalescent model that allows for the transmission of multiple lineages between hosts. With a bottleneck assumption many dated phylogenetic trees would not permit the overlaying of a transmission tree under our stochastic branching model.

Here, we assume that the within-host pathogen population size q(τ) grows linearly:


(24) q(τ)=κ+λτ,

where *τ* is the time since the host was infected. Should κ=0 all lineages will coalesce by the host’s infection time. We could adopt alternative population models, so long as they are integrable.

The likelihood of the phylogenetic tree conditional on the set of transmissions is calculated by taking the product of the likelihood of each *subtree* for each host. The subtree of any host *j* is formed by taking the parts of the phylogenetic tree assigned (colored) by host *j*. Each subtree is rooted at the host’s infection time $x^{j}$, with the number of roots being the number of lineages transmitted to the host. Leaves correspond to observations of the host and transmissions to the hosts included offspring, noting that each transmission may contribute multiple leaves (transmitting multiple lineages).

Let $v_{j}^{m}$, $m=1,\ldots ,M_{j}$ be the times leaves are added within the subtree of host *j*, and let $u_{j}^{n}$, $n=1,\ldots ,N_{j}$ be the coalescence times, supposing $N_{j}>0$. Then we define the number of extant lineages at time *t* as


(25) $$L_{j}(t)=\sum_{m=1}^{M_{j}}I(v_{j}^{m}\geq t)-\sum_{n=1}^{N_{j}}I(u_{j}^{m}>t),$$

so that if *t* is the time of a coalescence, $L_{j}(t)$ is the number of lineages that could have coalesced. Denoting $\tau_{j}=t-x^{j}$, the phylogenetic likelihood contribution from each host is then


(26) LP∣Tj(θP)=exp(−∫0∞(Lj(xj+τj)2)1q(τj)dτj)×∏n=1Nj1q(ujn−xj),

and the full phylogenetic likelihood conditional on transmission tree T is given by the product


(27) $$p(P|T,\theta_{P})=\prod_{\;j=1}^{N}L_{P|T}^{j}(\theta_{P}).$$

Let $w_{j}^{k}$, k=0,…,K be the ordered set of root, leaf, and coalescence times, with $w_{j}^{0}=x^{j}$. Let $L_{j}^{k}$ be the number of lineages in the interval $\left(w_{j}^{k-1},w_{j}^{k}\right)$. The integral in the exponent can then be partitioned accordingly


(28) ∫0∞(Lj(xj+τj)2)1q(τj)dτj=∑k=1n∫wjk−1−xjwjk−xj(Ljk2)1q(τj)dτj.

For the linear growth model, these terms are then


(29) ∫wjk−1−xjtjk−xj(Ljk2)1q(τj)dτj=(Ljk2)λ(log(κ+λ(wjk−xj))−log(κ+λ(wjk−1−xj))).

Phylogenetic tree simulation is described in supplementary text S2, Supplementary Material online.

### Inference

Inference is undertaken using reversible-jump MCMC (Green 1995). We iterate through the following update steps:

1. Update the transmission model parameters according to $p(\theta_{T}|T)$.
2. Update the coalescent model parameters according to $p(\theta_{P}|P,T)$.
3. Update the transmission tree according to $p(T|P,\theta_{T},\theta_{P})$.

Steps 1 and 2 are performed using multivariate Gaussian random walks, conditional on the current transmission and phylogenetic trees. The scale and covariance in each case is determined using the accelerated shaping and scaling algorithm of Spencer (2021) with target acceptance a=0.234 and forgetting sequence f(n)=⌊0.5n⌋.

In Step 3, we randomly select from 3 proposals that update the transmission tree conditional on the current model parameters: an add proposal for adding a new transmission to the current transmission tree, a remove proposal for removing a transmission, and a local move proposal for moving a transmission within the bounds set by its upstream and downstream transmissions. The add and remove proposals form a reversible pair that change the dimension of the model, whereas the local move proposal is its own reverse and maintains the dimension of the model. Each proposal ensures that the new transmission tree is compatible with the phylogenetic tree. For instance, observations from a single host cannot be split among multiple hosts when adding a transmission. Likewise, observations from different hosts cannot be assigned to the same host when removing a transmission. Full details including the acceptance probabilities for each proposal are provided in supplementary text S3, Supplementary Material online.

Step 3 makes relatively small changes to the transmission tree with each update. Additionally, the computational cost is relatively cheap as we only need to evaluate the likelihood contributions from the 1 or 2 affected hosts. Consequently, it is beneficial to perform Step 3 multiple times in each scan, in order to improve the mixing of the MCMC. In general, we find that performing O(N) Step 3 updates in each scan works well, where *N* is the number of primary observations.

### Implementation

We implemented the methods above into a new R package called TransPhyloMulti which extends TransPhylo. TransPhyloMulti is available at https://github.com/DrJCarson/TransPhyloMulti. This repository also contains all the code and data needed to reproduce all results shown in this paper. The R package ape was used to store, manipulate, and visualize phylogenetic trees (Paradis and Schliep 2019).

## Supplementary Material

msad288_Supplementary_Data
