# Supplementary material for: Inference of Infectious Disease Transmission through a Relaxed Bottleneck Using Multiple Genomes Per Host
Source: Mol Biol Evol. 2024 Jan 3;41(1):msad288. doi: 10.1093/molbev/msad288 (PMC10798190; doi:10.1093/molbev/msad288)
Supplement: msad288_Supplementary_Data [file msad288_supplementary_data.pdf]

Supplementary Material

# Inference of infectious disease transmission using multiple genomes per host

Jake Carson[1,2,3], Matt Keeling[1,2,3], David Wyllie[4], Paolo Ribeca[4], Xavier Didelot[2,3,5]

[1] Mathematics Institute, University of Warwick, Coventry CV4 7AL, United Kingdom

[2] School of Life Sciences, University of Warwick, Coventry CV4 7AL, United Kingdom

[3] Zeeman Institute for Systems Biology and Infectious Disease Epidemiology Research (SBIDER), University of Warwick, Coventry CV4 7AL, United Kingdom

[4] UK Health Security Agency, London NW9 5EQ, United Kingdom

[5] Department of Statistics, University of Warwick, Coventry CV4 7AL, United Kingdom

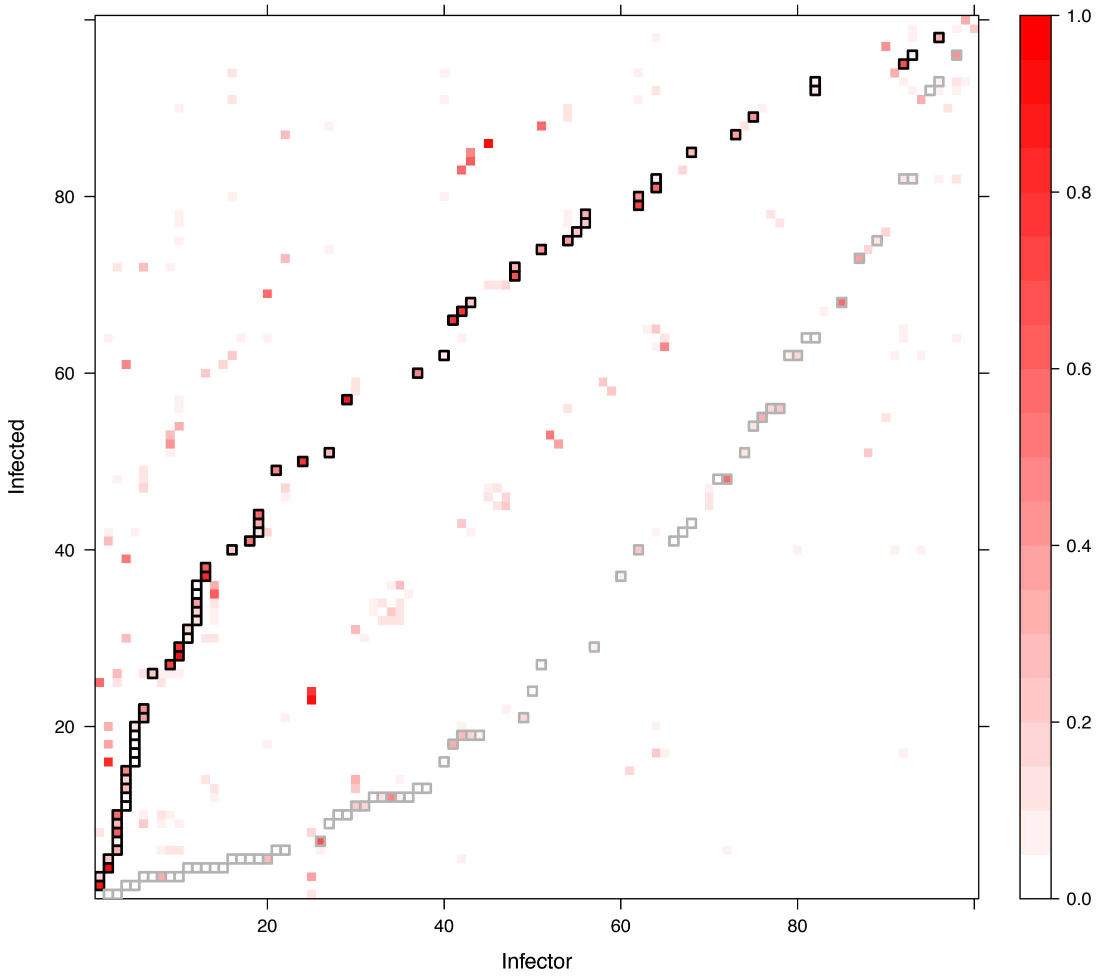


Figure S1: TransPhyloMulti: Posterior probability estimates of transmissions from an infector (row) to an infected host (column) for a simulated dataset with one observation per host. The black squares show the true transmissions in the simulated dataset. The gray squares show the reverse relationship, switching the true infector and infected hosts.

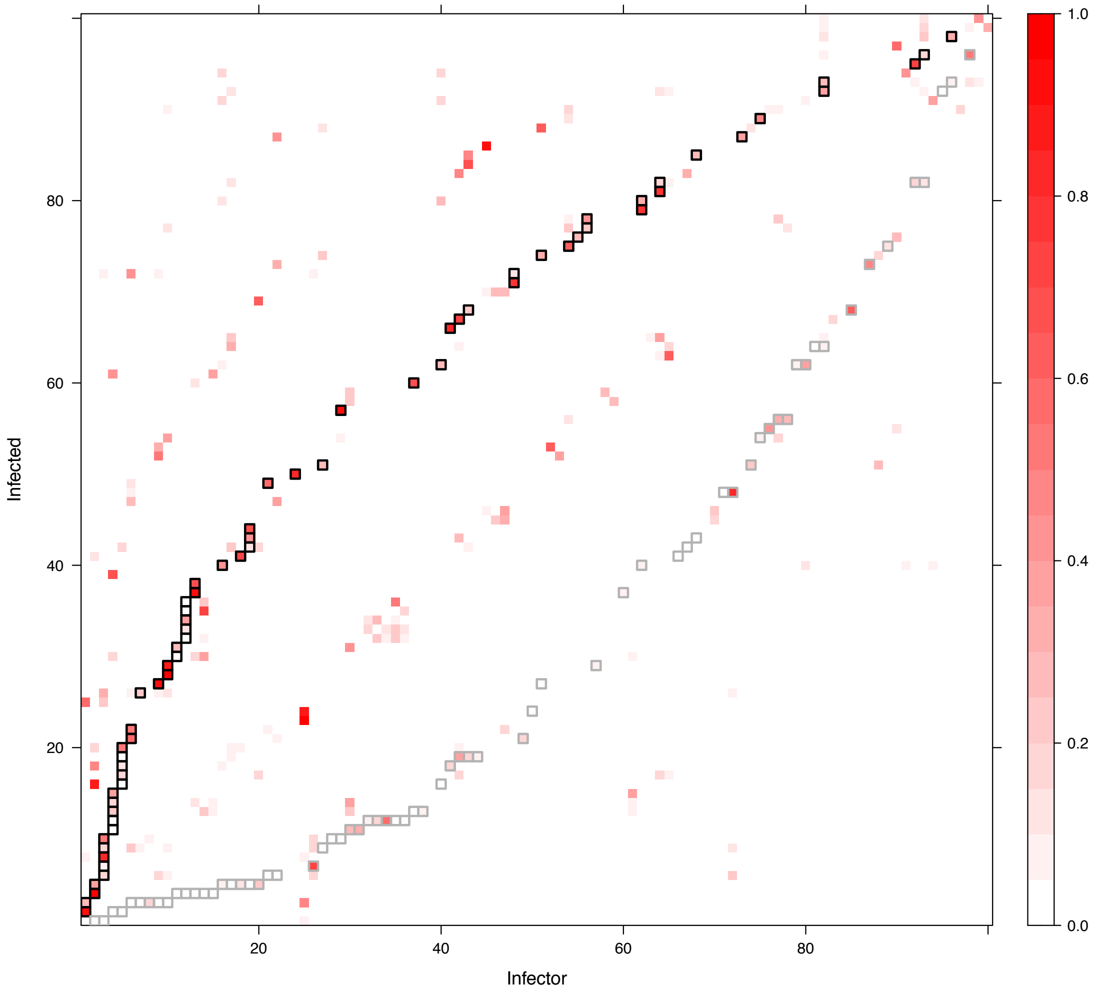


Figure S2: TransPhylo: Posterior probability estimates of transmissions from an infector (row) to an infected host (column) for a simulated dataset with one observation per host. The black squares show the true transmissions in the simulated dataset. The gray squares show the reverse relationship, switching the true infector and infected hosts.

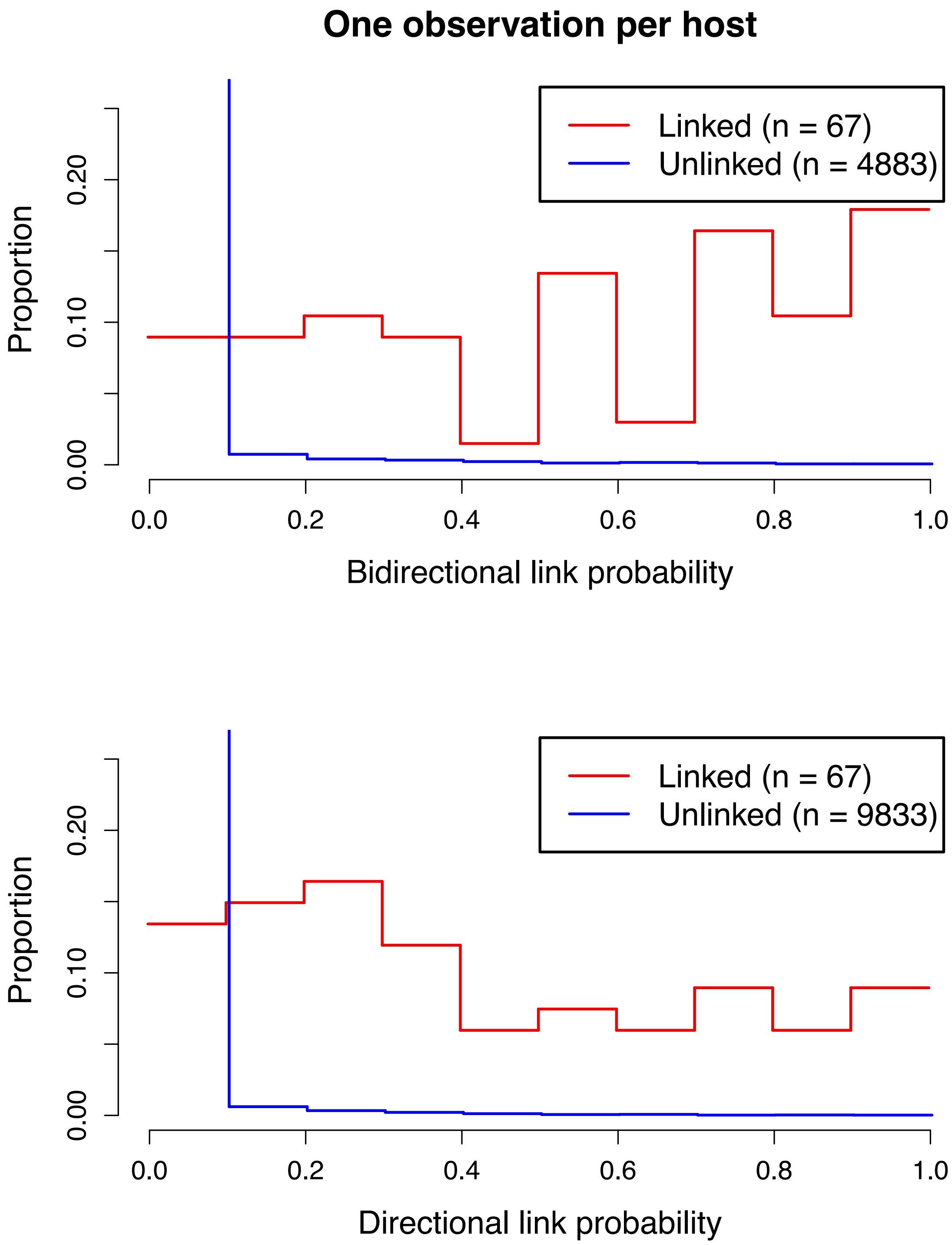


Figure S3: Distribution of posterior link probabilities inferred in the simulation studies with one observation per host using TransPhylo. The top plots show bidirectional link probabilities in which the roles of infector and infected host may switch, the bottom plots show the directional link probabilities in which the infector and infected host must be correctly inferred. The red lines relate to pairs of individuals for which a transmission link exists, and the blue lines relate to pairs of individuals that are not linked.

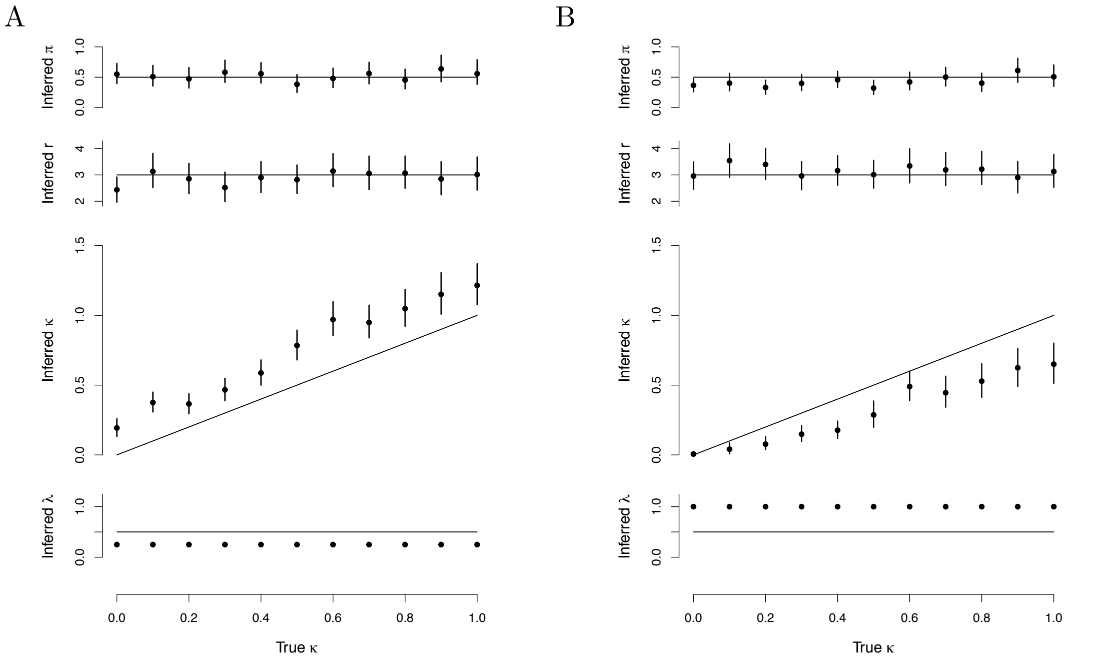


Figure S4: Posterior parameter estimates for the multiple simulations study with misspecified population growth rate $\lambda$. Vertical bars show 95% central credible intervals, and solid circles show posterior means. Horizontal and diagonal lines show the values used for simulations. (A) $\lambda$ is fixed at half the true value. (B) $\lambda$ is fixed at double the true value.

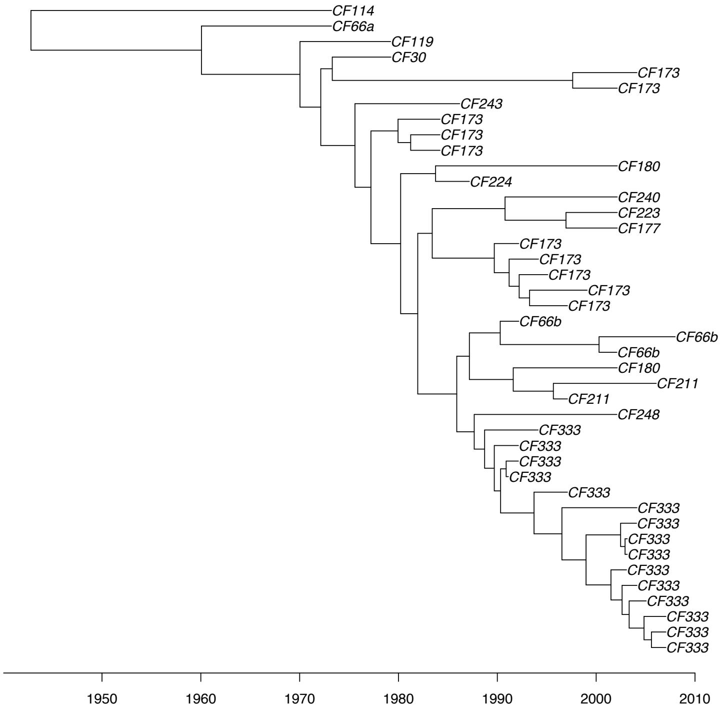


Figure S5: Dated tree used as input of the *P. aeruginosa* analysis.

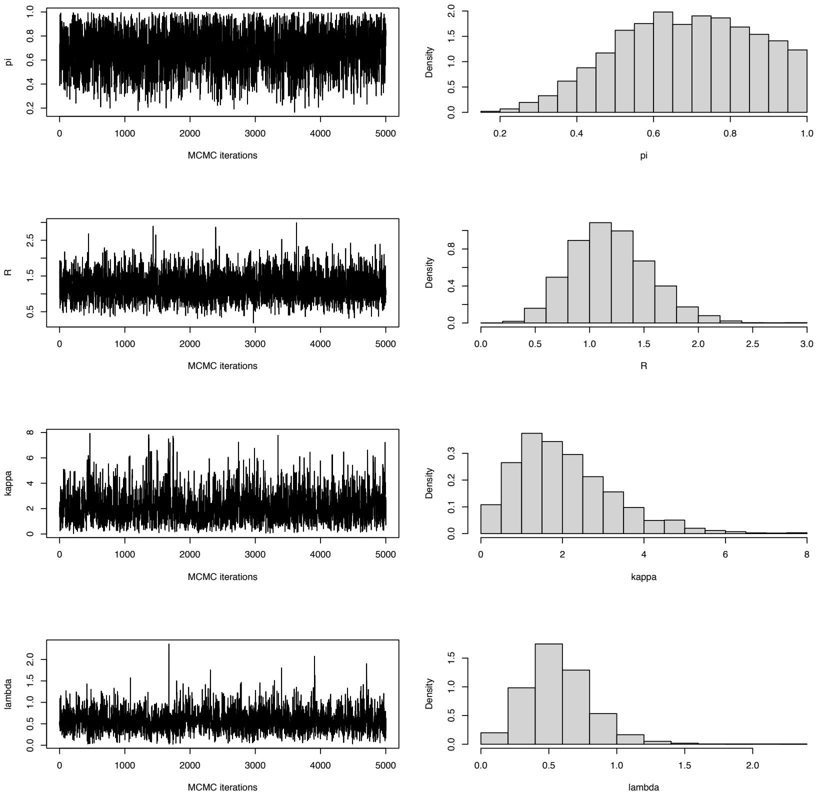


Figure S6: Parameter estimates in the *P. aeruginosa* analysis.

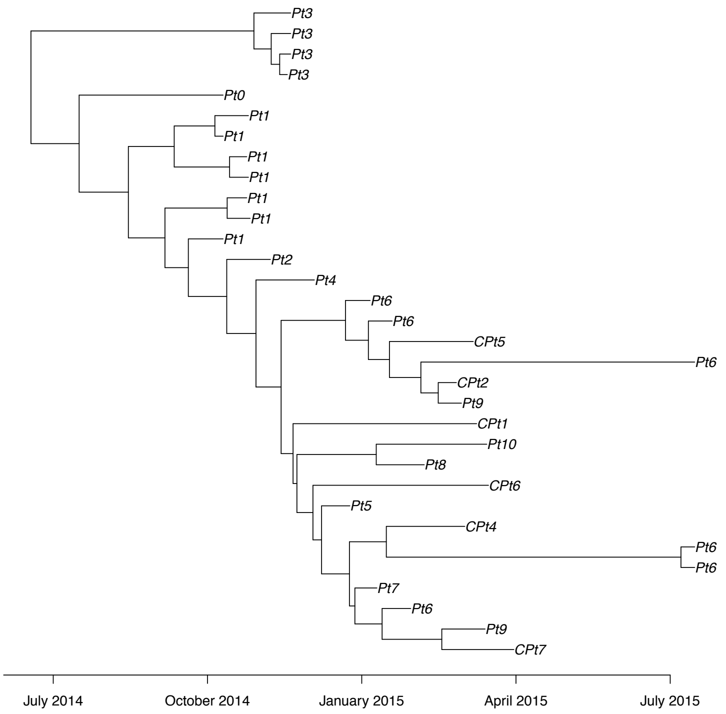


Figure S7: Dated tree used as input of the *K. pneumoniae* analysis.

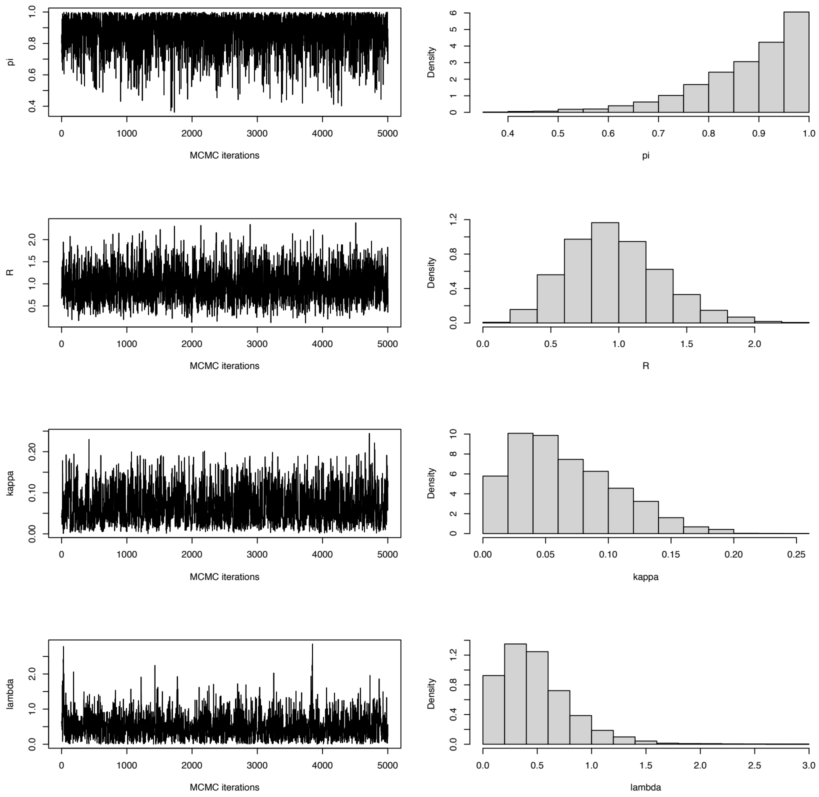


Figure S8: Parameter estimates in the *K. pneumoniae* analysis.

# Supplementary Text S1 Transmission tree simulation

There are multiple ways to simulate transmission trees in the ongoing outbreak scenario. One option is to fix the observation cut-off time $T$ and follow the steps used in the likelihood derivation in Equations (14) to (20). This is presented in Algorithm S1. Alternatively, an upper bound may be set for either the number of observed hosts or the number of observations, with $T$ being a simulated outcome. The transmission tree is simulated from the stochastic branching model, from the earliest infected host to latest, until this relevant bound is exceeded. This induces a temporary cut-off time $\tilde{T}$. Simulation then continues so long as there are hosts infected earlier than $\tilde{T}$ for which the number of observations and number of offspring have not been generated. Finally, a suitable value for $T$ is generated that returns the correct number of observed hosts / observations. When bounding by the number of observed hosts $T$ acts as a cut-off time for primary observations only, whereas when bounding by the number of observations $T$ acts as a cut-off time for both primary and secondary observations. Further details are given in Algorithms S2 and S3.

**Algorithm S1** Simulation of a transmission tree in an ongoing outbreak scenario with cut-off time $T$

Initialise root time $x^1$, $j \leftarrow 1$, $h \leftarrow 1$.
**while** $j \leq h$ **do**
  With probability $\frac{\varpi(x^j)}{1-\omega(x^j)}$ set $S^j \leftarrow 1$, otherwise set $S^j \leftarrow 0$.
  **if** $S^j = 1$ **then**
    Sample primary observation time from $\sigma(t - x^j)$.
    Sample the number of secondary observations according to $\beta(b)$ and their times according to $\rho(\tau_{1:b})$.
    Update vectors $y$, $z$, $H_y$, and $H_z$, censoring any observation times exceeding $T$.
    Sample number of included offspring $d^j \sim p(d \mid x^j)$.
  **else**
    Sample number of included offspring $d^j \sim p(d \mid x^j)$, $d > 0$.
  **end if**
  **for** $g = 1, ..., d^j$ **do**
    Set $h \leftarrow h + 1$ and set $A^h = j$.
    Sample infection time

$$x^h \sim \frac{(1 - \omega(t))\gamma(t - x^j)}{1 - \bar{\omega}(x^j)}, \qquad x^j < t < T.$$

  **end for**
  Set $j \leftarrow j + 1$.
**end while**

**Algorithm S2** Simulation of a transmission tree in an ongoing outbreak scenario with maximum number of observed hosts $N$

Initialise root time $x^1$, $j \leftarrow 1$, $h \leftarrow 1$, $n \leftarrow 0$, $\tilde{T} \leftarrow \infty$.
**while** $j \leq h$ **do**
with probability $\pi$ set $S^j \leftarrow 1$, otherwise set $S^j \leftarrow 0$.
**if** $S^j = 1$ **then**
Propose primary observation time from $\sigma(t - x^j)$.
Update number of observed hosts $n \leftarrow n + 1$.
**if** $n > N + 1$ **then**
Set $\tilde{T}$ as the time of the $(N+1)$'th primary observation, ordered earliest to latest.
**end if**
Propose number of secondary observations according to $\beta(b)$ and their times according to $\rho(\tau_{1:b})$.
Update vectors $y$, $z$, $H_y$, and $H_z$, censoring any observations from hosts with a primary observation time later than $\tilde{T}$.
**end if**
Sample number of offspring $k^j \sim \alpha(k)$.
**for** $g = 1, ..., k^j$ **do**
Propose infection time $\tilde{t} \sim \gamma(t - x^j)$.
**if** $\tilde{t} < \tilde{T}$ **then**
Set $h \leftarrow h + 1$ and set $A^h = j$.
Set $x^h \leftarrow \tilde{t}$.
**end if**
**end for**
**end while**
Set $T$ as a time between the $N$'th and $(N+1)$'th primary observation.
Censor any observations from hosts with a primary observation time later than $T$, updating $y$, $z$, $H_y$, and $H_z$ accordingly.
Discard excluded hosts, updating $x$ and $A$ accordingly.

**Algorithm S3** Simulation of a transmission tree in an ongoing outbreak scenario with maximum number of observations $N$

Initialise root time $x^1$, $j \leftarrow 1$, $h \leftarrow 1$, $n \leftarrow 0$, $\tilde{T} \leftarrow \infty$.
**while** $j \leq h$ **do**
  with probability $\pi$ set $S^j \leftarrow 1$, otherwise set $S^j \leftarrow 0$.
  **if** $S^j = 1$ **then**
    Propose primary observation time from $\sigma(t - x^j)$.
    Propose number of secondary observations according to $\beta(b)$ and their times according to $\rho(\tau_{1:b})$.
    Update vectors $y$, $z$, $H_y$, and $H_z$, censoring any observation times exceeding $\tilde{T}$.
    Update number of observations $n$.
    **if** $n > N + 1$ **then**
      Set $\tilde{T}$ as the time of the $(N + 1)$'th observation, ordered earliest to latest.
    **end if**
  **end if**
  Sample number of offspring $k^j \sim \alpha(k)$.
  **for** $g = 1, ..., k^j$ **do**
    Propose infection time $\tilde{t} \sim \gamma(t - x^j)$.
    **if** $\tilde{t} < \tilde{T}$ **then**
      Set $h \leftarrow h + 1$ and set $A^h = j$.
      Set $x^h \leftarrow \tilde{t}$.
    **end if**
  **end for**
**end while**
Set $T$ as a time between the $N$'th and $(N + 1)$'th observation.
Censor any observations later than $T$, updating $y$, $z$, $H_y$, and $H_z$ accordingly.
Discard excluded hosts, updating $x$ and $A$ accordingly.

## Supplementary Text S2 Phylogenetic tree simulation

The subtree for each host is simulated using repeated application of inverse transform sampling. Assume that at time $t$ there are $L$ extant lineages in a host infected at time $x^j$. We wish to sample the next coalescence time $u_j^*$, and so we solve

$$
\begin{aligned}
\nu &= 1 - \exp\left(-\int_{u_j^* - x^j}^{t - x^j} \binom{L}{2} \frac{1}{\kappa + \lambda\tau} \mathrm{d}\tau\right) \\
&= 1 - \left(\frac{\kappa + \lambda(u_j^* - x^j)}{\kappa + \lambda(t - x^j)}\right)^{\frac{\binom{L}{2}}{\lambda}}.
\end{aligned} \tag{30}
$$

Thus, we sample $\nu \sim \mathcal{U}(0, 1)$ and propose

$$
u_j^* = x^j + \frac{1}{\lambda}\left((1-\nu)^{\frac{\lambda}{\binom{L}{2}}}(\kappa + \lambda(t - x^j)) - \kappa\right). \tag{31}
$$

Should additional leaves be added at time $v_j^m$, where $u_j^* < v_j^m < t$, then the proposed coalescence time is discarded, and the next coalescence time is proposed by setting $t = v_j^m$ and $L = L + 1$. If $u_j^* < x^j$ then the coalescence time is discarded, and the $L$ lineages are assumed to have been transmitted to the host. Otherwise, we accept the proposed coalescence time and randomly select a pair of lineages to coalesce. We then propose the next coalescence time by setting $t = u_j^*$ and $L = L - 1$. The complete algorithm is presented in Algorithm S4.

**Algorithm S4** Simulation of a host subtree

Initialise $t \leftarrow v_j^M$; $L \leftarrow 1$; $m \leftarrow M$; $r \leftarrow 0$
**while** $t > x^j$ **do**
  Propose coalescence time

$$u_j^* = x^j + \frac{1}{\lambda}\left((1-\nu)^{\frac{\lambda}{\binom{L}{2}}}(\kappa + \lambda(t - x^j)) - \kappa\right)$$

  **if** $u_j^* > v_j^{m-1}$ **then**
    Set coalescence time $r \leftarrow r + 1$; $u_j^r \leftarrow u_j^*$; $L \leftarrow L - 1$; $t \leftarrow u_j^*$
  **else**
    $m \leftarrow m - 1$; $L \leftarrow L + 1$; $t \leftarrow v_j^m$
  **end if**
**end while**
**for** $n = 1, ..., r$ **do**
  Reverse coalescence times $u_j^n \leftarrow u_j^{r+1-n}$
**end for**

# Supplementary Text S3 Metropolis Hastings proposals for updating the transmission tree

## Add proposal

In the add proposal a new transmission is added to the transmission tree. We can interpret this proposal as sampling the following components:

1. Sampling the infector of the new host from the current hosts.
2. Sampling which of the infector's offspring become the offspring of the new host.
3. If the infector is observed under the current transmission tree, determine whether those observations are assigned to the infector or the new host.
4. Sampling a transmission time (the infection time of the new host).

Note that the new host must be included in order to form a consistent transmission tree, requiring at least one offspring or observation. Additionally, the phylogenetic tree constrains which set of offspring can be infected by the new host, and the set of times over which the transmission can occur. The combined sampling information also determines how coalescent events are divided between the two hosts.

Firstly, we sample the infector uniformly from the set of current hosts. We then consider how a transmission event can be added to the infector's subtree. The division of the offspring and observations between the two hosts is determined by the branch or branches over which the transmission occurs. Consequently, the discrete parts of our proposal corresponds to sampling this set of branches.

Assume that an infector's subtree contains B branches with start times $t_1^b$ and end times $t_2^b$ for $b \in \{1, ..., B\}$. Let $\Omega$ denote a sequence of all possible combinations of branches, such that $|\Omega| = 2^B - 1$ is the total number of possible combinations. For a given combination $j \in \{1, ..., 2^B - 1\}$ define $\hat{t}_1^j = \max(t_1^b \mid b \in \Omega^j)$ and $\hat{t}_2^j = \min(t_2^b \mid b \in \Omega^j)$, so that $\Delta^j = \max(0, \hat{t}_2^j - \hat{t}_1^j)$ is the length of time over which the branches overlap. Finally, let $V^j = \Delta^j \mathbb{I}^j_{\text{compatible}}$, where $\mathbb{I}^j_{\text{compatible}} = 1$ if adding a transmission to branches $\Omega^j$ results in a compatible transmission tree, and $\mathbb{I}^j_{\text{compatible}} = 0$ otherwise. Whilst this seems like an expensive set of calculations, note that most branch combinations do not overlap and we can easily restrict our calculations to those that do. Additionally, some branches can be pre-grouped where such groupings are necessary to form a compatible transmission tree. A combination $j$ is sampled according to the probabilities $V^j / \sum_k V^k$. Once a combination of branches has been sampled, we sample the transmission time uniformly in the interval $(\hat{t}_1^j, \hat{t}_2^j)$.

As an example, consider adding a transmission to the coloured phylogenetic tree depicted in Figure S9A with Host 1 as the infector. We will refer to the newly added host as Host P. There are three branches within the subtree and one possible combination of two branches with non-zero overlap. This gives us four possible placements of the new transmission:

1. Between the infection time of Host 1 and the coalescent, on the branch leading to the coalescent.

2. Between the coalescent and the infection time of Host 2, on the branch leading to the infection of Host 2.

3. Between the coalescent and the infection time of Host 3, on the branch leading to the infection of Host 3.

4. Between the coalescent and the infection time of Host 2, on the two branches leading to the infections of Host 2 and Host 3.

These different options are shown in Figure S9B. Note that placement 1 and 4 describe the same transmission tree. Host 1 infects Host P, and Host P infects Hosts 2 and Host 3. However, the number of transmitted lineages, and in which host the coalescent lies, depends on the exact placement. Evaluating $V^1 = 1$, $V^2 = 1$, $V^3 = 2$, $V^4 = 1$ gives us the probability vector $(0.2, 0.2, 0.4, 0.2)$ for sampling the placement.

If we assume that the current transmission tree contains $N_{\text{tot}}$ hosts, the proposal density is therefore

$$\begin{aligned} q_{\text{add}}(\mathcal{T}' \mid \mathcal{T}) &= \frac{1}{N_{\text{tot}}} \cdot \frac{V^j}{\sum_k V^k} \cdot \frac{1}{\Delta^j} \\ &= \frac{1}{N_{\text{tot}} \sum_k V^k}, \end{aligned} \tag{32}$$

where $\mathcal{T}'$ is the proposed transmission tree. Assuming that $N'_{\text{rem}}$ transmissions are removable in the proposed tree, the reverse density is

$$q_{\text{rem}}(\mathcal{T} \mid \mathcal{T}') = \frac{1}{N'_{\text{rem}}}. \tag{33}$$

The Metropolis Hastings acceptance ratio is therefore

$$\alpha(\mathcal{T}' \mid \mathcal{T}) = \min\left(1, \frac{p(\mathcal{P} \mid \mathcal{T}', \theta_P) p(\mathcal{T}' \mid \theta_T) N_{\text{tot}} \sum_k V^k}{p(\mathcal{P} \mid \mathcal{T}, \theta_P) p(\mathcal{T} \mid \theta_T) N'_{\text{rem}}}\right), \tag{34}$$

as the determinant of the Jacobian is 1. Note that we only need to calculate contributions to the phylogenetic and transmission tree likelihoods from the affected hosts as the remaining terms will cancel. More formally, let $h_1$ be the sampled infector in the current transmission tree, and $h'_1$ and $h'_2$ be the infector and new infected host in the proposed transmission tree, then

$$\alpha(\mathcal{T}' \mid \mathcal{T}) = \min\left(1, \frac{\mathcal{L}^{h'_1}_{P|T'}(\theta_P) \mathcal{L}^{h'_2}_{P|T'}(\theta_P) \mathcal{L}^{h'_1}_{T'}(\theta_T) \mathcal{L}^{h'_2}_{T'}(\theta_T) N_{\text{tot}} \sum_k V^k}{\mathcal{L}^{h_1}_{P|T}(\theta_P) \mathcal{L}^{h_1}_{T}(\theta_T) N'_{\text{rem}}}\right), \tag{35}$$

where $\mathcal{L}_{P|T'}$ and $\mathcal{L}_{T'}$ are likelihoods computed under the proposed transmission tree.

## Remove proposal

In the remove proposal we first determine which transmissions can be removed such that the new transmission tree remains consistent with the phylogenetic tree. In particular this means

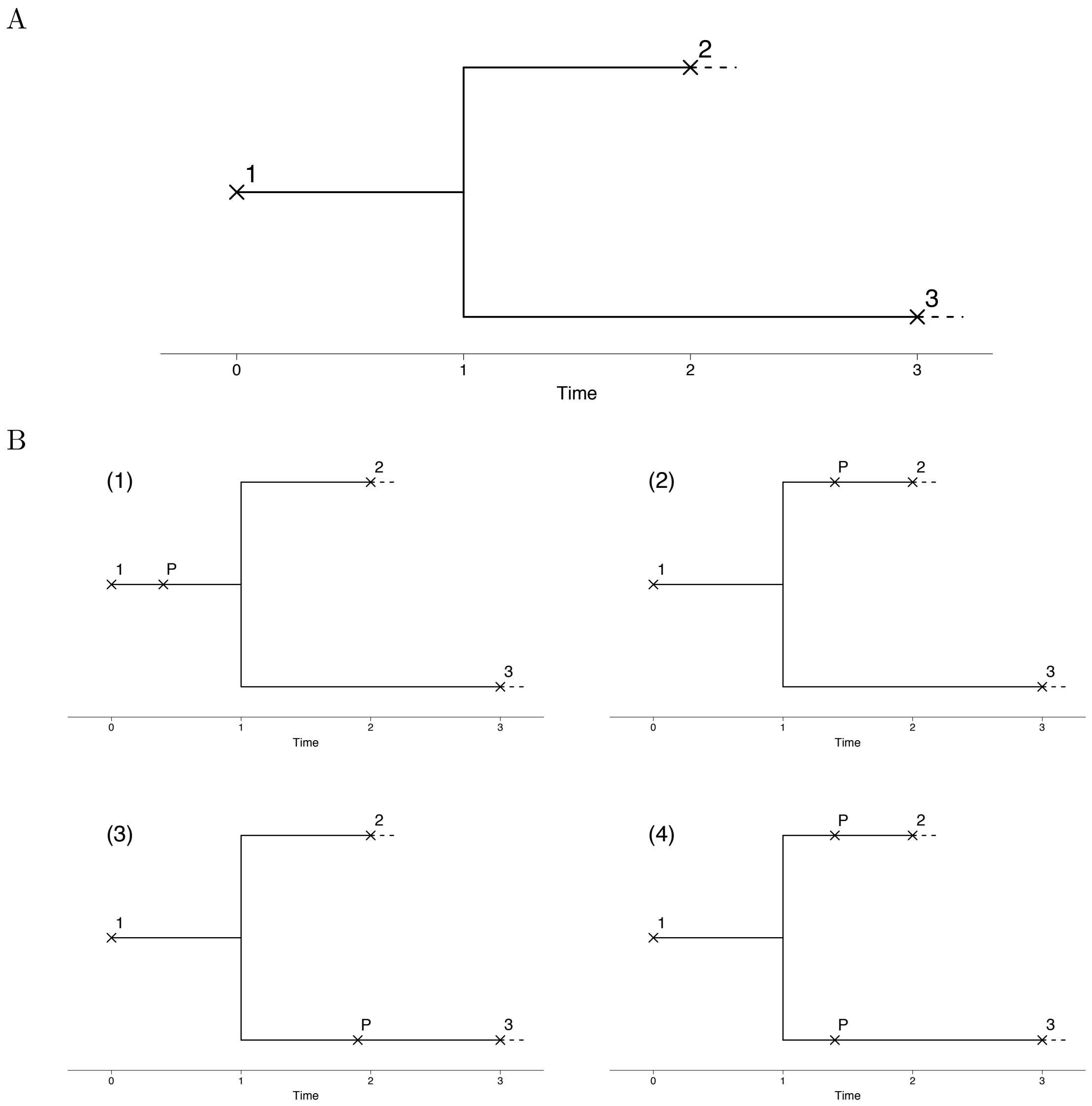


Figure S9: (A) Part of a coloured phylogenetic tree focused on host 1. Transmissions are indicated by × and labelled by the infected host number. (B) Examples of the three topologies when adding a transmission. Transmissions are indicated by × and labelled by the infected host number. Under the existing transmission tree, Host 1 infects both Host 2 and Host 3. When adding a transmission, another host is added, denoted as Host P.

that we can not remove transmissions between two observed hosts, since these observations would then be assigned to a single host. From this subset, and excluding the root transmission, a single transmission is sampled at random and removed.

If $N_{\text{rem}}$ transmissions are removable in the current transmission tree, the proposal density is

$$q_{\text{rem}}(\mathcal{T}' \mid \mathcal{T}) = \frac{1}{N_{\text{rem}}}. \tag{36}$$

Letting $N'_{\text{tot}}$ be the total number of hosts in the proposed transmission tree, the reverse move corresponds to the add proposal, so that

$$q_{\text{add}}(\mathcal{T} \mid \mathcal{T}') = \frac{1}{N'_{\text{tot}} \sum_k V'^k}, \tag{37}$$

where $V'^k$ are the time intervals described in the add proposal, constructed under the proposed transmission tree. The Metropolis Hastings acceptance ratio is therefore

$$\alpha(\mathcal{T}' \mid \mathcal{T}) = \min\left(1, \frac{\mathcal{L}^{h'_1}_{P|T'}(\theta_P)\mathcal{L}^{h'_1}_{T'}(\theta_T) N_{\text{rem}}}{\mathcal{L}^{h_1}_{P|T}(\theta_P)\mathcal{L}^{h_2}_{P|T}(\theta_P)\mathcal{L}^{h_1}_{T}(\theta_T)\mathcal{L}^{h_2}_{T}(\theta_T) N'_{\text{tot}} \sum_k V'^k}\right). \tag{38}$$

where $h_1$ and $h_2$ are the infector and infected hosts of the sampled transmission event in the current transmission tree, and $h'_1$ is the combined host in the proposed transmission tree.

## Local move proposal

Under the local move proposal we aim to move a transmission with minimal changes to the transmission tree topology. This can also be seen as a remove-add proposal, where we first remove a transmission, and then add a new transmission nearby. The remove step is similar to the remove proposal, but any transmission can be sampled. We are no longer concerned about whether removing a transmission results in an inconsistent transmission tree, as we can ensure consistency when the transmission is re-added. If the root transmission is sampled, a new root time is proposed using a Gaussian random walk. Otherwise, the selected transmission is removed, in essence combining two hosts into one, which is then treated as the infector in the add proposal. Note that if the transmission changes branches this may reverse the infector and infected host of the sampled transmission.

The local move proposal is symmetric, and so the acceptance probability is

$$\alpha(\mathcal{T}' \mid \mathcal{T}) = \min\left(1, \frac{\mathcal{L}^{h'_1}_{P|T'}(\theta_P)\mathcal{L}^{h'_2}_{P|T'}(\theta_P)\mathcal{L}^{h'_1}_{T'}(\theta_T)\mathcal{L}^{h'_2}_{T'}(\theta_T)}{\mathcal{L}^{h_1}_{P|T}(\theta_P)\mathcal{L}^{h_2}_{P|T}(\theta_P)\mathcal{L}^{h_1}_{T}(\theta_T)\mathcal{L}^{h_2}_{T}(\theta_T)}\right). \tag{39}$$

where $h_1$ and $h_2$ are the infector and infected hosts in the current transmission tree, and $h'_1$ and $h'_2$ in the proposed transmission tree. Note that if the root transmission is sampled, $h_1 = h'_1 = 0$ and the corresponding likelihood terms are excluded.
